# AZD7648 is a potent and selective DNA-PK inhibitor that enhances radiation, chemotherapy and olaparib activity

**DOI:** 10.1038/s41467-019-12836-9

**Published:** 2019-11-07

**Authors:** Jacqueline H. L. Fok, Antonio Ramos-Montoya, Mercedes Vazquez-Chantada, Paul W. G. Wijnhoven, Valeria Follia, Neil James, Paul M. Farrington, Ankur Karmokar, Sophie E. Willis, Jonathan Cairns, Jenni Nikkilä, David Beattie, Gillian M. Lamont, M. Raymond V. Finlay, Joanne Wilson, Aaron Smith, Lenka Oplustil O’Connor, Stephanie Ling, Stephen E. Fawell, Mark J. O’Connor, Simon J. Hollingsworth, Emma Dean, Frederick W. Goldberg, Barry R. Davies, Elaine B. Cadogan

**Affiliations:** 1 Bioscience, Oncology R&D, AstraZeneca, Cambridge, UK; 2 Mechanistic Biology and Profiling, Discovery Sciences, Oncology R&D, AstraZeneca, Cambridge, UK; 3 Translational Science, Oncology R&D, AstraZeneca, Cambridge, UK; 4 Quantitative Biology, Discovery Science, Oncology R&D, AstraZeneca, Cambridge, UK; 5 Medicinal Chemistry, Oncology R&D, AstraZeneca, Cambridge, UK; 6 DMPK, Oncology R&D, AstraZeneca, Cambridge, UK; 7 Oncology Business Unit, AstraZeneca, Cambridge, UK; 8 Oncology Translational Medicine Unit, Early Clinical Development, Oncology R&D, AstraZeneca, Cambridge, UK

**Keywords:** Drug development, Clinical pharmacology

## Abstract

DNA-dependent protein kinase (DNA-PK) is a critical player in the DNA damage response (DDR) and instrumental in the non-homologous end-joining pathway (NHEJ) used to detect and repair DNA double-strand breaks (DSBs). We demonstrate that the potent and highly selective DNA-PK inhibitor, AZD7648, is an efficient sensitizer of radiation- and doxorubicin-induced DNA damage, with combinations in xenograft and patient-derived xenograft (PDX) models inducing sustained regressions. Using ATM-deficient cells, we demonstrate that AZD7648, in combination with the PARP inhibitor olaparib, increases genomic instability, resulting in cell growth inhibition and apoptosis. AZD7648 enhanced olaparib efficacy across a range of doses and schedules in xenograft and PDX models, enabling sustained tumour regression and providing a clear rationale for its clinical investigation. Through its differentiated mechanism of action as an NHEJ inhibitor, AZD7648 complements the current armamentarium of DDR-targeted agents and has potential in combination with these agents to achieve deeper responses to current therapies.

## Abstract (web summary)

DNA-dependent protein kinase (DNA-PK) plays a major role in the DNA damage response upon double-strand break formation. Here, the authors show that the DNA-PK inhibitor AZD7648, enhances the activity of radiotherapy, chemotherapy and the PARP inhibitor olaparib in multiple mouse tumour models.

## Introduction

Tumour cells manage their inherent genomic instability through the detection and repair of DNA lesions by a process termed the DNA damage response (DDR), which triggers intracellular signalling events,  regulates processes including cell cycle progression and promotes DNA repair. This process also affords the tumour cell some protection from therapy-induced DNA damage^1^. Inability to accurately repair DNA lesions can lead to increased genomic instability, senescence or cell death. Most, if not all, cancers will have lost one or more aspects of DDR capability; therefore, inhibiting components of the DDR process may be an effective therapeutic strategy for the treatment of cancer^2^.

DNA double-strand breaks (DSBs) are considered the most deleterious of DNA lesions. Pathological DNA DSBs can be caused by DNA replication defects, inappropriate nuclease activity or exogenous sources such as ionizing radiation (IR) or topoisomerase II inhibitors^3^. There are two major canonical mechanisms for the repair of DNA DSBs; homologous recombination repair (HRR) and non-homologous end joining (NHEJ). The HRR pathway is utilized during the S and G2 phases of the cell cycle when a sister chromatid is available to be used as a DNA template. In the absence of a sister chromatid, cells can utilize other repair pathways such as NHEJ. The repair of DNA DSB generated by IR and topoisomerase II inhibitors mainly requires DNA-dependent protein kinase (DNA-PK), a complex that consists of the KU heterodimers (KU70 and KU80) and the DNA-dependent protein kinase catalytic subunit DNA-PKcs^4–6^. DNA-PKcs, a member of the phosphoinositide 3 lipid kinase (PI3K)-related protein kinase (PIKK) family, becomes activated when bound to KU and orchestrates this repair by phosphorylating factors including those of the NHEJ machinery, such as Artemis and XRCC4 (ref. ^7^), as well as the DNA damage marker histone H2AX on Ser139 (generally referred to as γH2AX)^8^, while its autophosphorylation (pSer2056) is important for DNA-end processing and accessibility^9,10^. The critical role of NHEJ in the repair of physiological DSBs generated during V(D)J recombination and class-switch recombination is well established^3^. In addition, DNA-PK has been found to have roles in other cellular processes, including modulation of chromatin structure, telomere maintenance and transcriptional regulation^11,12^. Besides these more established roles, DNA-PKcs has also been recently implicated in the cellular DNA replication stress response (RSR) through its phosphorylation of RPA32 and its effects on the ATR–CHK1 axis^13–15^.

The importance of DNA-PK activity in repairing pathological DNA DSBs is illustrated by studies showing that DNA-PKcs genetic deficiencies sensitize cells to IR and other DSB-inducing agents^16–18^. This highlights the suitability of using a DNA-PK inhibitor as a combination partner with IR and topoisomerase II inhibitors such as doxorubicin and etoposide. Indeed, there have been efforts in developing small-molecule DNA-PK inhibitors for these purposes in the past^19^. Older-generation DNA-PK inhibitors such as NU7441, NU7026 and KU-0060648, while useful probe compounds, were limited by poor selectivity against other PIKKs or structurally related PI3Ks. The challenges of developing selective DNA-PK inhibitors are further exemplified by LY3023414 and CC-115, two compounds currently in clinical development but with dual activity against both DNA-PK and the mammalian target of rapamycin (mTOR), a PIKK family member^20,21^. More recently, the newer-generation DNA-PK selective compounds VX-984 and M3814 (refs. ^22–24^) have progressed to clinical development in combination with liposomal doxorubicin and IR, respectively (trial identifiers: VX-984; NCT02644278, M3814; NCT02516813).

While the potential for DNA-PK inhibitors as combinatorial agents with radiation and topoisomerase II inhibitors is well established, the therapeutic opportunities for combinations with other DDR-targeted agents have not been explored. Studies showing that loss of ataxia-telangiectasia mutated (ATM) kinase, a cardinal kinase in DDR, or deficiency in MutS homologue 3 (MSH3) increases cellular sensitivity to DNA-PK inhibitor (KU-0060648) treatment^25–27^ suggest that there may be opportunities for combination with other inhibitors of DDR, particularly if those effects are significantly enhanced by tumour-specific DDR deficiencies such as in ATM signalling.

In this article, we describe a potent and selective DNA-PK inhibitor, AZD7648, that enhances the efficacy of both IR and doxorubicin. In addition, we identify PARP inhibitors as a potential combination partner for DNA-PK inhibitors, using olaparib, which is currently approved for a number of indications in breast and ovarian cancer^28,29^. With all three combinations, tumour regression at tolerated doses in vivo was observed, and regulatory approval has now been obtained to progress these combinations in the clinic (trial identifier: NCT03907969).

## Results

### Discovery of a potent and selective DNA-PK inhibitor

**Fig. 1:**
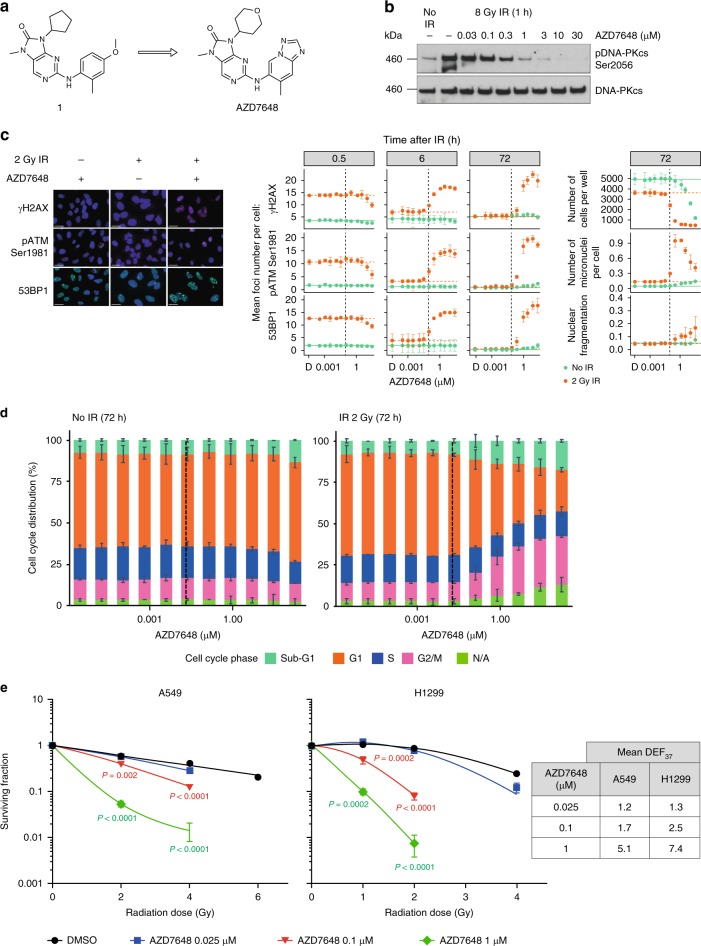
AZD7648 is a potent radiosensitizer in vitro. **a** Optimization of screening hit 1 into the potent and selective DNA-PK inhibitor, AZD7648. **b** Western blot analysis of A549 cells treated with 8 Gy IR ± 1 h pre-treatment with increasing concentrations of AZD7648. **c** High-content imaging analysis of indicated DNA damage markers in A459 cells. Cells were treated with 2 Gy IR ± AZD7648 pre-treatment (1 h) and immunofluorescently stained at indicated time points. Graphs represent mean foci, number of cells, micronuclei per cell or relative nuclear fragmentation from two independent experiments (*n* = 2, ±SEM). Black dotted lines indicate AZD7648 cellular IC_50_ concentration (91 nM). Representative images are shown for 2 Gy IR ± 1.25 μM AZD7648 at 72 h (scale bars, 25 μM). D represents DMSO vehicle-treated controls. **d** Cell cycle analysis was performed based on DAPI staining intensity of nuclei detected by imaging analysis. Graphs represent cell cycle distribution (percentage population) from a representative experiment (*n* = 2). N/A (non-assigned) represents cell populations where signal intensities exceeded the threshold to accurately determine the cell cycle phase. Dotted lines indicate AZD7648 cellular IC_50_ concentration (91 nM). **e** Colony formation assays performed with A549 or NCI-H1299 cells treated with an ionizing radiation dose response ± AZD7648. Graphs represent mean surviving fraction normalized to the single-agent activity of AZD7648. Data were fitted to the linear quadratic model (mean ± SD (*n* = 2); unpaired *t*-test where *P* ≤ 0.05 is significant). Mean dose enhancement factor values (DEF_37_) are shown. DAPI 4′,6-diamidino-2-phenylindole, DMSO dimethyl sulfoxide, SD standard deviation, SEM standard error of the mean

While the ATP-binding pocket of DNA-PKcs is considered druggable by small molecules^19,30^, it can be challenging to identify and optimize the selectivity of inhibitors versus both the structurally related PI3Ks and other PIKK family members, such as ATM kinase, ataxia telangiectasia and Rad3-related protein (ATR), and mTOR. Consequently, we screened the AstraZeneca corporate collection to identify potent DNA-PK inhibitors with good selectivity versus PI3Kα. The resulting screening hit 1 (Fig. 1a) was optimized to increase potency while improving physicochemical properties and pharmacokinetics (PK). The optimized compound, AZD7648, potently inhibits DNA-PK kinase activity in a biochemical assay (50% inhibitory concentration [IC_50_] = 0.6 nM). In a panel of 397 kinases screened with a compound concentration of 1 μM by ThermoFisher (SelectScreen: Kinase Profiling Service), only DNA-PK, PI3Kα, PI3Kδ and PI3Kγ showed inhibition of >50%. Biochemical selectivity ratios were then assessed at ThermoFisher with dose–response data, and AZD7648 was found to be >100 times more selective for DNA-PK versus PI3Kα (p110 alpha/p85 alpha or p110 alpha/p55 gamma) and PI3Kδ (p110 delta/p85 alpha), and 63 times more selective versus PI3Kγ (p110 gamma). In IR-treated A549 cells, AZD7648 potently inhibits DNA-PKcs autophosphorylation at Ser2056 (IC_50_ = 91 nM; Fig. 1b, Table 1). Furthermore, AZD7648 showed >90-fold cellular selectivity over ATM, ATR, mTOR, and three PI3K isoforms (PI3Kα, PI3Kβ and PI3Kδ) and >10-fold selectivity over PI3Kγ (Table 1). By comparison, KU-0060648, NU7441 and M3814 all exhibited <10-fold selectivity in at least one secondary pharmacology target (Supplementary Table 1). Consequently, AZD7648 is a suitable probe molecule for establishing the effects of pharmacologically inhibiting DNA-PK activity.

**Table 1:** Cellular pharmacology of AZD7648

Assay target	IC_50_, *n* (pIC_50_ ± 2 SEM)
DNA-PKcs	91.3 nM, 13 (7.04 ± 0.15)
ATM	17.93 μM, 7 (4.75 ± 0.15)
ATR	>29.77 μM, 5 (<4.53 ± 0.005)
PI3Kα	>8.03 μM, 6 (5.07 ± 0.2)
PI3Kβ	>30 μM, 5 (<4.53)
PI3Kγ	1.37 μM, 7 (5.86 ± 0.35)
PI3Kδ	>30 μM, 7 (<4.53)
mTOR	>30 μM, 4 (<4.53)

Results are presented as geometric mean IC_50_ and number of repeats, *n*. The mean pIC_50_ ± 2 SEM are described in parentheses. pIC_50_, −log (IC_50_)

### AZD7648 is a potent radiosensitizer in vitro and in vivo

DNA DSB-inducing therapeutic modalities, such as IR, are rational combination partners for DNA-PK inhibitors. Many factors involved in the DDR are recruited to and/or phosphorylated at DNA damage sites upon IR treatment and form distinctive DNA damage-induced nuclear foci detectable by immunofluorescence^31^. Formation of γH2AX, 53BP1 and ATM phosphorylated on Ser1981 (pATM) foci, as well as micronuclei, are consistent cellular response markers of DSBs caused by IR^32–34^. To evaluate the radio-sensitizing characteristics of AZD7648, we investigated the kinetics of DNA damage following AZD7648 treatment in IR-treated A549 non-small-cell lung cancer (NSCLC) cells using these markers. NSCLC cell lines and xenograft models were chosen as IR is a standard-of-care in this disease. IR treatment of vehicle-treated control cells led to γH2AX, 53BP1 and pATM foci formation, which recovered to baseline by 6 h (Fig. 1c). Similar results were observed in conditions whereby cells received 1 h pre-treatment of AZD7648 at concentrations below the IC_50_ (<91 nM). However, at concentrations greater than the AZD7648 IC_50_, γH2AX, 53BP1 and pATM foci persisted at least until 72 h after IR treatment, suggesting that DNA repair was delayed by DNA-PK inhibition with AZD7648. To confirm these findings, we performed neutral comet assays and observed that the IR-induced nuclear γH2AX, 53BP1 and pATM foci indeed represent physical DSBs that persist in presence AZD7648 (Supplementary Fig. 1). These data correlate with the appearance of micronuclei and a prolonged G2/M cell cycle arrest at later time points, further confirming persistence of DNA damage when combining IR with AZD7648 (Fig. 1c, d). The combination of AZD7648 with IR led to a concentration-dependent decrease in clonogenic survival of A549 and NCI-H1299 NSCLC cells (Fig. 1e, Supplementary Table 2). Together these data demonstrate in vitro that AZD7648 leads to the persistence of DNA damage following IR, resulting in G2/M DNA damage checkpoint activation, genome instability and reduced cellular survival.

**Fig. 2:**
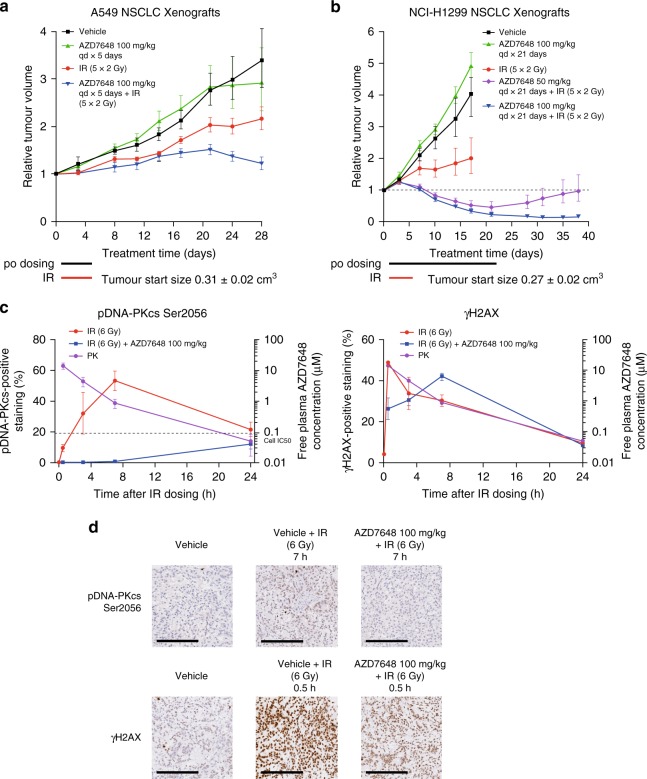
AZD7648 is a potent radiosensitizer of tumours in vivo. **a** A549. AZD7648 induces tumour growth inhibition in combination with IR in A549 xenografts (nude mice, vehicle and combination *n* = 11, IR *n* = 12, AZD7648 *n* = 9, geometric mean ± SEM). **b** NCI-H1299. AZD7648 induces tumour regression in combination with IR in NCI-H1299 xenografts (nude mice, vehicle *n* = 13, IR and AZD7648 100 mg kg^−1^ combination *n* = 11, AZD7648 single agent and 50 mg kg^−1^ combination *n* = 12, geometric mean ± SEM). For **a** and **b**, corresponding mouse bodyweights and statistical analysis can be found in Supplementary Fig. 2A, B and Supplementary Table 3A. To assess tumour growth inhibition, one-tailed, two-sample, *t*-test with unequal variances was used and for tumour regression, one-sample *t*-test analysis. **c**, **d** Pharmacodynamic modulation of DNA-PK biomarkers and pharmacokinetics (PK) of AZD7648 after dosing of AZD7648 and IR in A549 xenografts. pDNA-PKcs Ser2056 and γH2AX were measured by immunohistochemistry (image scale bars, 200 μm; nude mice, 0 h *n* = 8, all treatments *n* = 5, mean ± SEM). Several comparisons between the IR and IR + AZD7648 treatments were found to be statistically significant using a one-way ANOVA. For pDNA-PKcs Ser2056: 0.5 h (*p* < 0.0001), 3 h (*p* = 0.0005) and 7 h (*p* < 0.0001). For γH2AX: 0.5 h (*p* = 0.05) and 7 h (*p* = 0.02)

When tested in vivo, we observed that AZD7648 administered orally at a tolerated dose of 100 mg kg^−1^ once daily (qd) further enhanced the response to fractionated IR (2 Gy for five consecutive days) in mice implanted with A549 xenografts (Fig. 2a). While tumours were insensitive to single-agent AZD7648 treatment, IR treatment alone induced tumour growth inhibition (TGI) by 50%, but the combination of AZD7648 with IR (AZD7648 followed by IR 1 h later in the first 5 days of the study) achieved 90% TGI. This in vivo radio-sensitization was shown to be dose-dependent in NCI-H1299 xenografts, where the combination (AZD7648 100 or 50 mg kg^−1^ qd × 21 days + IR 2 Gy qd × 5 days) achieved 85% or 55% tumour regression compared with 60% TGI induced by IR alone (Fig. 2b). To understand AZD7648 pharmacodynamic modulation in vivo, we studied the phosphorylation status of three markers of DNA-PK activity, pDNA-PKcs Ser2056 (refs. ^9,35^), γH2AX^8,36^, and pRPA32 Ser4/Ser8 (ref. ^15^), by either immunohistochemistry (IHC) or western blot analysis. A549 xenografts treated with a single dose of IR (6 Gy) induced increases in pDNA-PKcs Ser2056 and γH2AX (Fig. 2c, d) which were abolished by a single dose of AZD7648 100 mg kg^−1^ (dosed 1 h before IR). Comparable with the in vitro results, the addition of AZD7648 delayed the normal reduction of γH2AX levels back to baseline relative to IR treatment alone. Similar results were observed with western blot analysis in NCI-H1299 xenografts treated with the combination (Supplementary Fig. 3). In this model, the increase in phosphorylation of DNA-PKcs at Ser2056, γH2AX and RPA32 at Ser4/Ser8 induced by IR was reduced by AZD7648, tracking with AZD7648 plasma concentration.

### AZD7648 increases sensitivity to doxorubicin

**Fig. 3:**
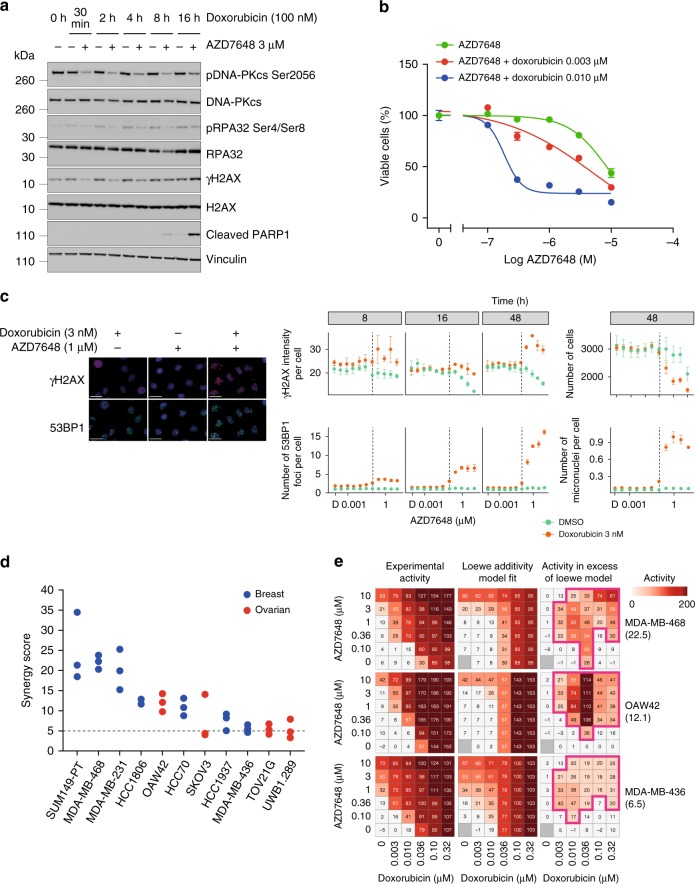
AZD7648 and doxorubicin have synergistic combination activity in breast and ovarian cancer cell lines. **a** Western blot analysis of OAW42 cells treated with doxorubicin (100 nM) ± AZD7648 (3 μM). **b** Concentration-dependent response to AZD7648 ± doxorubicin was measured by a Live/Dead assay. Graphs represent percentage viable cells ± SD following 5 days’ treatment relative to DMSO vehicle-treated controls from a representative experiment (*n* = 3). **c** High-content imaging analysis of indicated DNA damage markers in OAW42 cells. Cells were treated with increasing AZD7648 concentrations ± doxorubicin (3 nM) and immunofluorescently stained at indicated time points. Graphs represent mean ± 2 SEM γH2AX signal intensity, number of 53BP1 foci, number of cells/well, or micronuclei per cell from three independent experiments. Representative images are shown for AZD7648 (1 μM) ± doxorubicin (3 nM) at 48 h (scale bars, 25 μm). ***D*** represents DMSO vehicle-treated controls. **d** Synergy scores for the AZD7648 and doxorubicin combination in a panel of four ovarian and seven breast cancer cell lines. Cells were treated for 5–7 days and viability was measured by the Live/Dead assay. A synergy score of >5 is indicative of synergistic activity. **e** Activity heatmaps from representative experiments for MDA-MB-468, OAW42 and MDA-MB-436 cells. Experimental activity heatmap represents growth inhibitory (0–100) and cytotoxic activity (100–200) following treatment. Loewe additivity model fit heatmap represents expected activity values for an additive combination. Concentrations where combination activity occurred in excess of the expected activity are boxed in pink. Synergy scores from the representative experiment are indicated in brackets

Doxorubicin is a topoisomerase II poison and DNA intercalator that generates DNA DSBs, making it another rational therapeutic combination partner for DNA-PK inhibitors^5^. Clinically, doxorubicin/DOXIL® is primarily used for the treatment of solid tumours, including ovarian and breast cancer, hence we chose in vitro and in vivo models to represent these tumour types. In an ovarian cancer cell line (OAW42) treated with doxorubicin, AZD7648 treatment downregulated pDNA-PKcs Ser2056, γH2AX Ser139 and pRPA32 Ser4/Ser8 phosphorylation at early time points (at 30 min, 2 h and 4 h) as detected by western blotting (Fig. 3a). At later time points (8 and 16 h), the combination resulted in increased levels of γH2AX and the apoptosis marker cleaved PARP1 compared with doxorubicin treatment alone (Fig. 3a). Indeed, the combination of AZD7648 and doxorubicin led to a concentration-dependent reduction in cell viability of OAW42 cells following 5 days of treatment (Fig. 3b). We next analysed the cellular response to DNA damage in this model following AZD7648 and doxorubicin combination treatment by immunofluorescence imaging. Total nuclear intensity of γH2AX was used instead of foci as an indicator of DNA damage, as the staining of γH2AX was too strong following doxorubicin (3 nM) treatment in the presence of AZD7648 to accurately determine foci numbers. We observed that the total nuclear intensity of γH2AX and number of 53BP1 foci and micronuclei increased in a time- and AZD7648-concentration-dependent manner (Fig. 3c). Altogether, these in vitro data suggest that combination treatment leads to increased DNA damage and genomic instability compared with either agent alone. Combination activity of AZD7648 and doxorubicin was further assessed across a panel of four ovarian and seven breast cancer cell lines, and synergistic growth inhibition activity (synergy score ≥5) was observed in all cell lines tested, based on the Loewe additivity model (Fig. 3d)^37^. Using three representative cell lines with a range of synergy scores, we observed growth inhibitory activity in excess of the data fit predicted by the Loewe model across the majority of the combination concentrations tested (Fig. 3e).

**Fig. 4:**
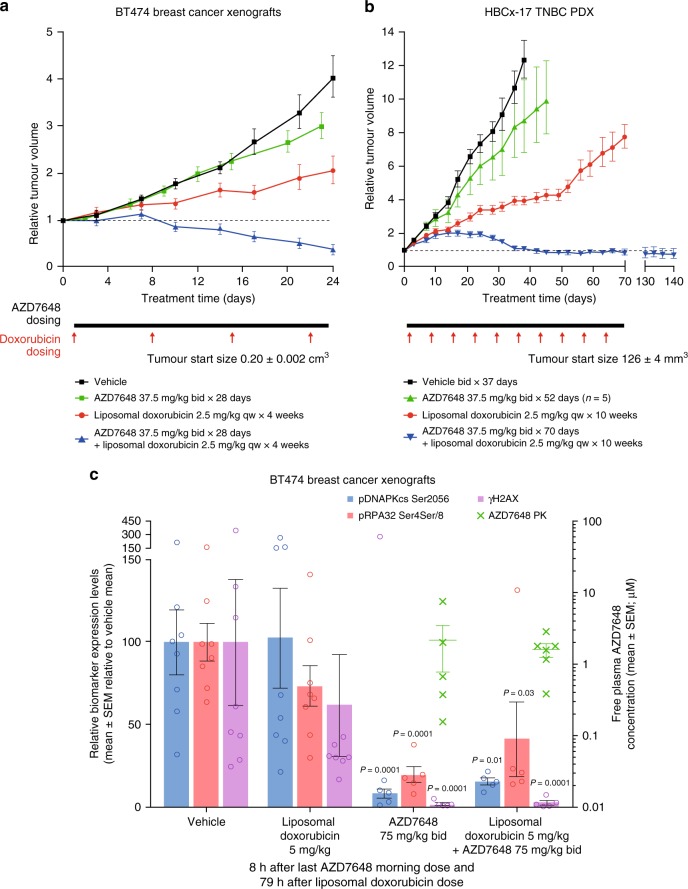
AZD7648 and liposomal doxorubicin synergize to inhibit tumour growth in vivo. **a** BT474. AZD7648 induces tumour regression in combination with liposomal doxorubicin in BT474 breast cancer xenografts (nude mice, vehicle and AZD7648 *n* = 12, liposomal doxorubicin *n* = 9, combination *n* = 11, geometric mean ± SEM). **b** AZD7648 induces tumour stasis in combination with liposomal doxorubicin in HBCx-17 TNBC PDX (nude mice, *n* = 10, geometric mean ± SEM). For **a** and **b**, corresponding mouse bodyweights and statistical analysis can be found in Supplementary Figs. 2C and 5A and Supplementary Table 3B. To assess tumour growth inhibition, a one-tailed, two-sample, *t*-test with unequal variances was used and for tumour regression, one-sample *t*-test analysis. **c** Pharmacokinetics of AZD7648 and pharmacodynamic modulation of DNA-PK biomarkers pDNA-PKcs Ser2056, pRPA32 Ser4/Ser8, and γH2AX after dosing of AZD7648 and liposomal doxorubicin in BT474 xenografts. Mice were dosed once with liposomal doxorubicin and with AZD7648 for 3 days. Samples were then collected 8 h after a last morning dose of AZD7648 on the fourth day. Measured by western blotting (nude mice, vehicle and liposomal doxorubicin *n* = 8, AZD7648 and combination *n* = 5, mean ± SEM). Significance assessed with two-sided *t*-tests performed on log-transformed data assuming unequal variance

When tested in vivo, dose-dependent TGI was observed in BT474 breast cancer xenografts treated with a range of tolerated AZD7648 doses (4, 12, 24 and 37.5 mg kg^−1^ bid × 28 days) and liposomal doxorubicin (2.5 mg kg^−1^ every week × 4 weeks) (Supplementary Fig. 4). AZD7648 at 37.5 mg kg^−1^ induced 20% TGI and doxorubicin induced 63% TGI, but the combination resulted in 77% regression (Fig. 4a). AZD7648 significantly reduced phosphorylation of DNA-PKcs at Ser2056, RPA32 at Ser4/Ser8 and the levels of γH2AX in the presence of doxorubicin (Fig. 4c). Combination benefit of AZD7648 and doxorubicin was also demonstrated in the triple-negative breast cancer (TNBC) patient-derived xenograft (PDX) model HBCx-17 (ATM WT, *TP53* mutant, *BRCA2* mutant, *CCNE* amplified, *CDKN2A* deleted), achieving 100% TGI while their respective single-agent treatments only induced 25% and 70% TGI (Fig. 4b). Altogether, the enhancement of IR and doxorubicin activity by AZD7648 accompanied by robust pharmacodynamic biomarker modulation in vitro and in vivo demonstrates the potential clinical utility for using these combination and gave us confidence to further explore other potential combination partners for AZD7648 in preclinical models.

### AZD7648 enhances the activity of PARP inhibitor olaparib

**Fig. 5:**
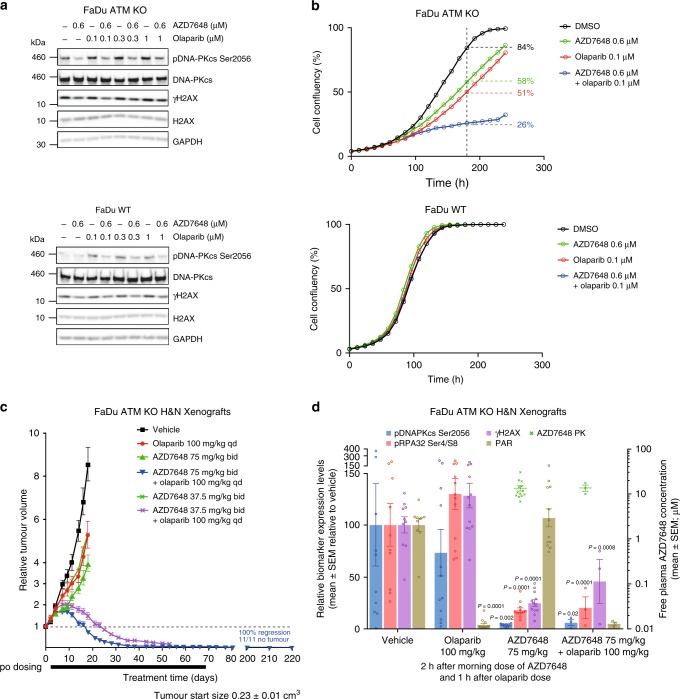
AZD7648 and olaparib combination has antiproliferative efficacy. **a** Western blot analysis of whole-cell lysates from FaDu WT or ATM KO cells treated with AZD7648 (0.6 μM), olaparib (0.1, 0.3 or 1 μM) or the combination for 24 hours. Both cell lines were run on the same blot. **b** Cell confluency of FaDu ATM KO and WT cells treated with AZD7648, olaparib or their combination. Graphs represent mean percentage cell confluency from a representative experiment (*n* = 4). Percentage values indicate final cell confluency at the indicated time point. **c** AZD7648 induces complete tumour regression in combination with olaparib in FaDu ATM KO xenografts (SCID mice, vehicle *n* = 6, olaparib *n* = 10, AZD7648 37.5 and 75 mg kg^−1^ single agent and combination at 37.5 mg kg^−1^ *n* = 8, combination at 75 mg kg^−1^ *n* = 11, geometric mean ± SEM). Corresponding mouse bodyweights and statistical analysis can be found in Supplementary Fig. 2E and Supplementary Table 3C. To assess tumour growth inhibition, one-tailed, two-sample, *t*-test with unequal variances was used and for tumour regression, one-sample *t*-test analysis. **d** Pharmacokinetics of AZD7648 and pharmacodynamic modulation of DNA-PK biomarkers after dosing of AZD7648 and olaparib in FaDu ATM KO xenografts. Mice were taken from the efficacy study described above (**c**) between days 11 and 18, and samples were collected 2 h after the first dose of AZD7648 (1 h after the dose of olaparib). Measured by western blotting (SCID mice, vehicle *n* = 10, olaparib *n* = 11, AZD7648 *n* = 12, combination *n* = 3, mean ± SEM). Significance assessed with two-sided *t*-tests performed on log-transformed data assuming unequal variance

PARP inhibitors are approved therapies for the treatment of a number of breast and ovarian cancer indications^28^. Central to their mode of action is the ability to trap PARP onto DNA at single-strand breaks, which in turn has the potential to stall replication forks and generate DNA DSBs when those replication forks collapse^38^. In our studies we chose to use breast and ovarian PDX models in line with clinical approval for olaparib. Although PARP inhibitors are currently used to treat tumours with *BRCA1/2*-deficiencies^28^, the loss of *BRCA1* or *BRCA2* in cell lines did not sensitize to AZD7648 monotherapy (Supplementary Fig. 6A), prompting us to seek an alternative genetic background to explore the potential for a PARP inhibitor and AZD7648 combination. *ATM*-deficient cells have been found to be especially sensitive to treatment with olaparib, a PARP inhibitor^39,40^, reportedly as a result of the loss of ATM activity in counteracting the toxic consequences of aberrant DNA-end joining at the broken replication forks^41^. Since it has been shown that the loss of *ATM* also sensitizes cancer cells to DNA-PK inhibitor treatment^25–27^, we sought to explore the effectiveness of the combination of olaparib and AZD7648 in *ATM*-deficient models preclinically. We utilized two isogenic cell line pairs to test this idea, namely the FaDu head and neck and the A549 NSCLC cell lines, where the *ATM* gene had been knocked out (KO) to enable comparison with their wild-type (WT) counterparts. We first confirmed that the ATM KO cell lines did not express *ATM* (Supplementary Fig. 7A) and that olaparib treatment led to an increase in DNA-PKcs autophosphorylation that was abrogated with AZD7648 treatment as had been previously reported^42^ (Fig. 5a, Supplementary Fig. 7B). We also confirmed that ATM KO cells demonstrated significantly greater sensitivity (>10-fold) to either AZD7648 or olaparib single-agent treatment compared with their respective isogenic WT cells (Supplementary Fig. 6B, C).

Using AZD7648 at 0.6 μM and olaparib at 0.1 μM (concentrations promoting near 50% of maximal inhibition of cell proliferation [GI_50_] or clonogenic survival [IC_50_] in FaDu ATM KO cells), we observed a marked combination effect on cell growth in both the FaDu and A549 ATM KO cells compared with the WT cells (Fig. 5b, Supplementary Fig. 7C). A greater reduction in cell viability was also observed for the combination treatment of AZD7648 and olaparib compared with the single agents across a larger panel of ATM WT cell lines, where synergy scores between 0 and 5 were achieved, indicative of combination benefit (Supplementary Fig. 7D). In vivo, when a combination of AZD7648 (75 mg kg^−1^ bid × 70 days) and olaparib (100 mg kg^−1^ qd × 70 days) was dosed to mice implanted with FaDu ATM KO xenografts, the treatment induced complete tumour regression, with 11 out of 11 mice showing no presence of tumour even 150 days after the combination treatment had stopped (Fig. 5c), whereas the combination treatment was not as efficacious in FaDu WT xenografts (Supplementary Fig. 8). Combination of olaparib with a lower dose of AZD7648 (37.5 mg kg^−1^ bid × 53 days) in FaDu ATM KO xenografts also induced effective tumour regression, albeit with a delay compared with the higher dose (Fig. 5c).

**Fig. 6:**
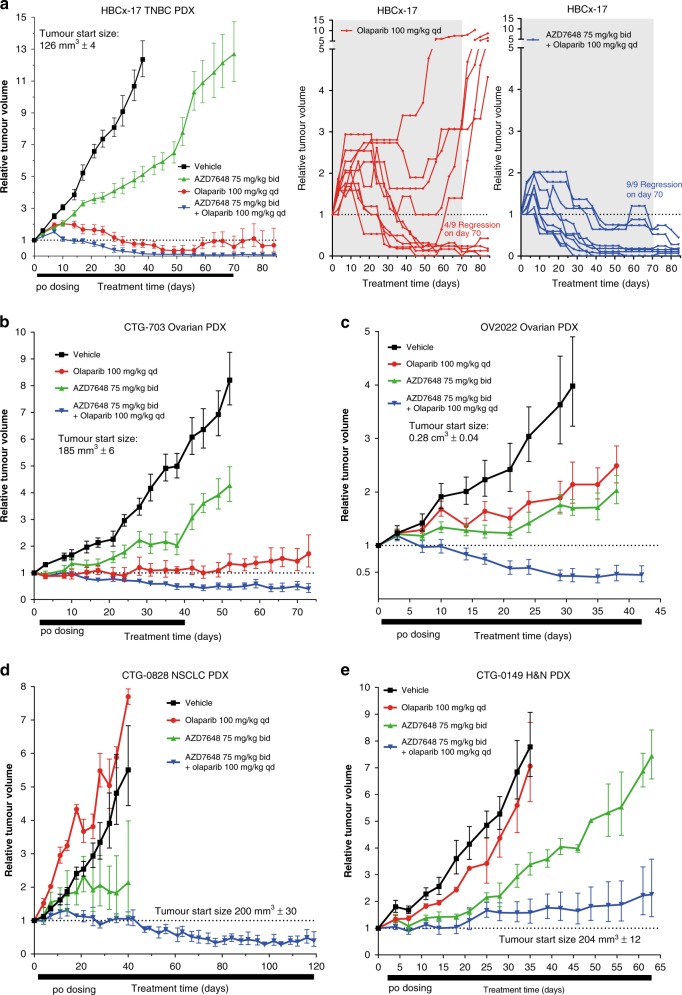
AZD7648 and olaparib combination has anti-tumour efficacy in PDX models. **a** AZD7648 induces tumour regression in combination with olaparib in HBCx-17 TNBC PDX (nude mice, *n* = 10; individual tumour spider plots for olaparib and combination groups). **b** AZD7648 induces tumour regression in combination with olaparib in CTG-703 ovarian PDX (nude mice, olaparib *n* = 4, other treatments *n* = 6). **c** AZD7648 induces tumour regression in combination with olaparib in OV2022 ovarian PDX (SCID mice, *n* = 8). **d** AZD7648 induces tumour regression in combination with olaparib in CTG-0828 NSCLC PDX (nude mice, vehicle *n* = 5, other treatments *n* = 3). **e** AZD7648 in combination with olaparib induces significant TGI in CTG-0149 H&N PDX (nude mice, vehicle *n* = 5, other treatments *n* = 3). All graphs represent geometric mean ± SEM. Corresponding mouse bodyweights and statistical analysis can be found in Supplementary Fig. 5B–F and Supplementary Table 3C. To assess tumour growth inhibition, one-tailed, two-sample, *t*-test with unequal variances was used and for tumour regression, one-sample *t*-test analysis

In the FaDu ATM KO xenograft model, AZD7648 was effective at reducing DNA-PK activity, measured using pDNA-PKcs Ser2056, γH2AX and pRPA32 Ser4/Ser8 (~80% reduction after 30 min 75 mg kg^−1^ dose of AZD7648), with recovery during the following 24 h closely tracking the pharmacokinetic profile of AZD7648, especially for pDNA-PKcs (Supplementary Fig. 9). Biomarker inhibition by AZD7648 was also observed in the presence of olaparib (Fig. 5d). The combination was also efficacious in the olaparib-sensitive PDX model HBCx-17, where the combination of AZD7648 (75 mg kg^−1^ bid × 70 days) and olaparib (100 mg kg^−1^ qd × 70 days) achieved 93% tumour regression, with nine out of nine tumours showing regression on day 70 (Fig. 6a). Meanwhile, olaparib single-agent treatment also induced tumour regression (42%), but only four tumours out of nine were regressed on the last day of dosing. AZD7648 single agent treatment, similar to the results with the FaDu ATM KO model, induced 65% TGI. The same combination treatment also showed to be very efficacious in four further PDX models (Fig. 6b–e); ovarian models CTG-703 (*ATM* WT, *BRCA1* mutant, *CHEK2* amplified, *TP53* mutant, *RB1* deleted, *XRCC3* deleted) and OV2022 (*ATM* WT, *TP53* mutant), NSCLC model CTG-0828 (*ATM* mutant, *FANCA* mutant, *NBN* mutant) and H&N model CTG-0149 (*ATM* WT, *TP53* mutant), achieving 60–70% tumour regression in the first three models and 90% TGI in the fourth model. In all four models the combination improved the effects above those obtained with olaparib, to which two models were insensitive (CTG-0828 and CTG-0149). In the other two models, OV2022 and CTG-703, olaparib did not induce regressions, but 65% and 100% TGI respectively. AZD7648 single-agent treatment induced 60–80% TGI in all models except on CTG-0828, where the data was too variable to assess the group effect. Together, these data suggest the potential for olaparib and AZD7648 combination benefit in genetic backgrounds beyond ATM deficiency.

### AZD7648 and olaparib combination treatment leads to genome instability and apoptosis

**Fig. 7:**
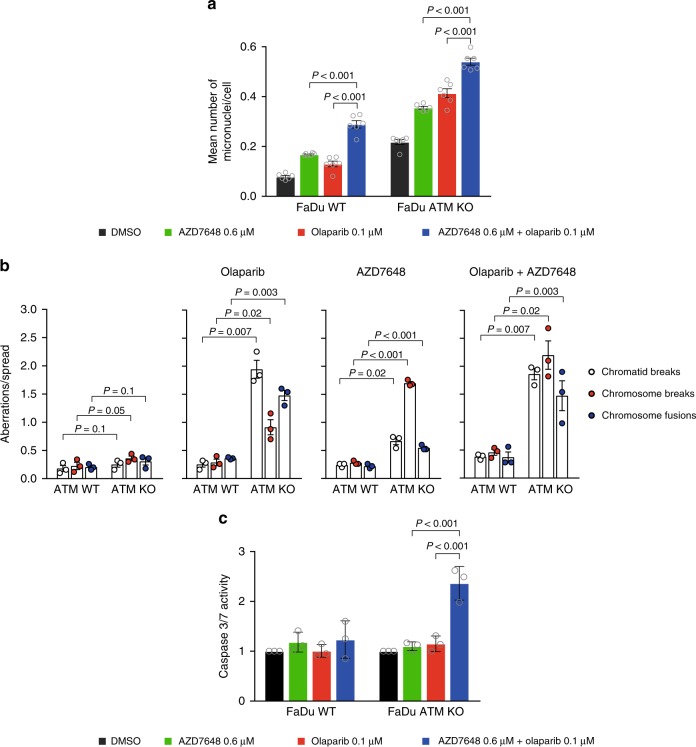
AZD7648 in combination with olaparib affects genome stability and induces apoptosis in ATM KO cells. **a** Frequency of micronuclei formation in FaDu WT and ATM KO cells following 72 h treatment. Data are shown as mean number of micronuclei per cell (mean ± SEM; *n* = 2; unpaired *t*-test, *P* ≤ 0.05 is significant). **b** Chromosomal aberrations in FaDu WT and ATM KO cells treated with olaparib (1 μM), AZD7648 (1 μM), or the combination for 48 h were detected by metaphase spread analysis. Chromatid breaks, chromosome breaks and chromosome fusions were quantified separately. Data are shown as mean aberrations per metaphase spread (mean ± SD; *n* = 3; paired *t*-test, *P* ≤ 0.05 is significant, 50 metaphase spreads/sample). **c** Caspase 3/7 activity of FaDu ATM KO and WT cells following 72 h treatment. Graph represents mean fluorescence levels normalized for total cell confluency and relative to the DMSO vehicle-treated control (mean ± SD; *n* = 3; two-way ANOVA, *P* ≤ 0.05 is significant)

We next sought to evaluate the mechanistic basis of the antiproliferative efficacy of the AZD7648 and olaparib combination. Since AZD7648 treatment reduced levels of γH2AX (Fig. 5a), we explored other means of assessing the consequence of DNA damage. A flow cytometry approach revealed that the combination of AZD7648 and olaparib induced an increase of the proportion of cells in the G2/M phase of the cell cycle (indicative of activation of the DNA damage checkpoint) that was abrogated by CHK1 inhibitor treatment, confirming that we indeed observed cell cycle arrest mediated by the ATR–CHK1 axis. However, the increase in the G2/M population of cells was not significantly different to that obtained from the single-agent treatments (Supplementary Fig. 7E). The combination did result in a concentration-dependent increase in the number of micronuclei formed in both the FaDu and A549 ATM KO cells compared with their WT equivalents and suggests that there is a potentiation of genome instability induced by the combination of olaparib and AZD7648, which is more pronounced in ATM KO cells (Fig. 7a, Supplementary Fig. 7F). To assess this further, and to allow for a more detailed interrogation of the kind of chromosomal aberrations that arise upon single agent or combination treatment, we performed metaphase spread analyses (Fig. 7b). Both olaparib and AZD7648 single-agent treatments were seen to increase the number of chromosomal aberrations, specifically in ATM KO cells, with AZD7648 mainly promoting the formation of chromosome breaks, which arise independently of replication status, while olaparib treatment produced replication-dependent chromatid breaks and chromosome fusions. These data therefore demonstrate a differential impact on genome stability of olaparib and AZD7648 single-agent treatments in the ATM KO cells. For the combination of the two agents, there was an increase in the total number of chromosomal aberrations compared with the respective single agents. To determine if the increased chromosomal instability translated into increased cell death, we analysed caspase 3/7 activity over time and observed significantly higher levels following AZD7648 and olaparib combination treatment compared with single-agent treatments (Fig. 7c, Supplementary Fig. 7G). Taken together, these data suggest that, compared with the single-agent treatments, the olaparib and AZD7648 combination promotes greater levels of genome instability, resulting in increased apoptosis, specifically in an *ATM*-deficient background.

### Intermittent schedules of AZD7648 are also efficacious

**Fig. 8:**
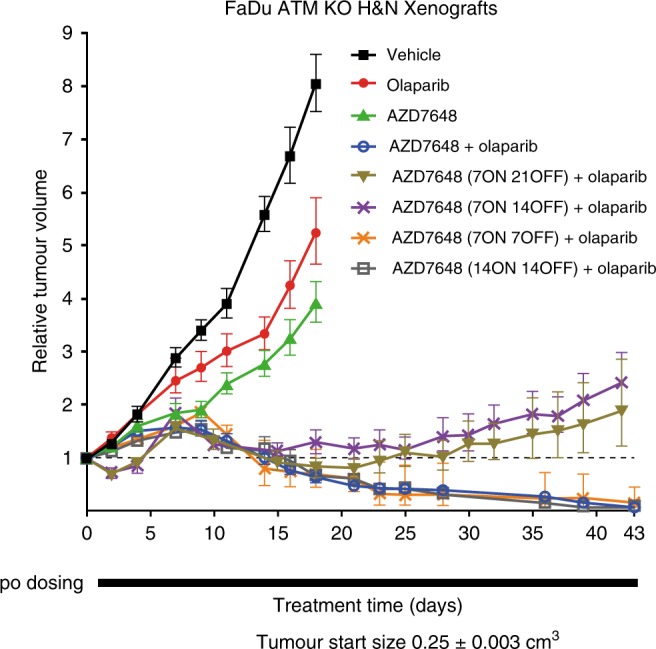
Anti-tumour effect of intermittent schedules of AZD7648 in combination with olaparib in FaDu ATM KO xenografts. Discontinuous schedules of AZD7648 increase the anti-tumour effects of olaparib in FaDu ATM KO xenografts. Doses: AZD7648 75 mg kg^−1^ bid; olaparib 100 mg kg^−1^ qd, dosed 1 h after AZD7648 dose (SCID mice, vehicle *n* = 13, olaparib *n* = 10, AZD7648 *n* = 8, continuous and 7ON 21OFF *n* = 6, 7ON 14OFF *n* = 7, 7ON 7OFF and 14ON 14OFF *n* = 5, geometric mean ± SEM). Corresponding mouse bodyweights and statistical analysis can be found in Supplementary Fig. 2G, Supplementary Table 3D. To assess tumour growth inhibition, one-tailed, two-sample, *t*-test with unequal variances was used and for tumour regression, one sample *t*-test analysis

In a clinical setting, where continuous administration of combined drug treatments is not always feasible due to overlapping toxicities, the ability to intermittently dose one agent while maintaining efficacy would have significant advantages. We used the FaDu ATM KO xenograft model to explore intermittent schedules of AZD7648 in combination with olaparib. Olaparib (100 mg kg^−1^ qd) was dosed continually and we introduced non-dosing periods (dosing holiday) for AZD7648 (Fig. 8). Four schedules were tested: 7ON 7OFF, 7ON 14OFF, 7ON 21OFF, and 14ON 14OFF. Of these four schedules, the 7ON 7OFF and 14ON 14OFF schedules achieved tumour regressions similar to those induced by the continuous combination schedule. The 7ON 14OFF and 7ON 21OFF schedules with longer holiday periods, while not achieving regressions, induced close to 100% TGI in the first 28 days of treatment, representing a significant improvement over continuous olaparib single-agent treatment (50% TGI). These results revealed the potential to have flexibility of AZD7648 dosing when combined with olaparib, which may prove valuable during its clinical development.

## Discussion

Targeting DDR dependencies of cancer cells has proven to be an effective therapeutic strategy in the case of PARP inhibitors^43^. The majority of DDR-targeted therapies currently in clinical development abrogate cell cycle checkpoints or modulate DNA DSB repair by targeting HRR^2^. The inhibition of NHEJ is an important additional strategy to realize the full potential of DDR-targeted therapies and their combinations clinically.

Small-molecule inhibitors of DNA-PK, a core mediator of NHEJ, have been disclosed previously, but their poor in vivo pharmacokinetic properties and/or lack of selectivity have limited their development^19^. Here, we report an inhibitor of DNA-PK kinase activity, AZD7648, with a considerably improved selectivity profile to previously reported inhibitors. We demonstrate that AZD7648 is both an efficacious combination partner with the standard-of-care therapies IR and doxorubicin, as well as report robust anti-tumour activity of DNA-PK inhibition in combination with the PARP inhibitor, olaparib, in preclinical cancer models.

The critical role of DNA-PK in mediating NHEJ repair of DNA DSBs caused by IR and other DSB-inducing agents is highlighted by reports showing that inhibiting DNA-PK or depleting other core NHEJ factors sensitizes cells to IR and topoisomerase II inhibitors such as doxorubicin and etoposide^4,5,17,18^. Ablating its kinase activity or mutating its autophosphorylation sites significantly impairs the ability of DNA-PKcs to promote effective NHEJ^4^. Consistent with these findings, both first- and second-generation small-molecule inhibitors of DNA-PK, have been shown to potentiate the activity of IR and etoposide^22–24,44–47^. In the case of NU7441 and KU-0060648 for example, the combination with etoposide led to greater anti-tumour efficacy in SW620 and MCF7 xenografts compared with their respective single-agent controls^44–47^. These results are in line with our study, which shows broad potentiation of doxorubicin by AZD7648 in panels of breast and ovarian cancer cell lines. Further studies in larger cell panels will be required to elucidate whether certain genetic backgrounds have increased susceptibility to the combination of AZD7648 with either doxorubicin or IR. As with the mechanism-of-action of NU7441 (ref. ^47^), the anti-tumour activity of AZD7648 in combination with IR or doxorubicin is associated with a decrease in DNA repair and an increase in DNA damage, demonstrated by elevated levels of γH2AX foci and an accumulation of the G2/M cell cycle population compared with the respective single-agent controls. This potentiation activity is reflected in our observations whereby AZD7648 in combination with IR or liposomal doxorubicin resulted in significant tumour growth inhibition and sustained regression in vivo.

PARP inhibitors, such as olaparib, are effective for the treatment of ovarian and breast cancers with deleterious *BRCA1* or *BRCA2* mutations^28,48^. Their clinical monotherapy activity is due to the unique ability of such molecules to trap PARP at single-stranded DNA breaks, leading to replication fork stalling and collapse, resulting in the generation of one-ended DNA DSBs^38^. Such DSBs can only be repaired faithfully by HRR that involves factors such as *BRCA1/2*, and therefore provides a mechanistic basis for the sensitivity of *BRCA1/2*-deficient cells to PARP inhibition^49,50^. The insupportable genomic instability caused by PARP inhibitor treatment in these cellular backgrounds are evident by the accumulation of DNA breaks, some of which are converted to chromosome fusions that are a consequence of aberrant DNA-end-joining events of one-ended DSBs through a process generally referred to as toxic NHEJ^42,51^.

A recent study shows that ATM counteracts toxic NHEJ at topotecan or olaparib-induced broken replication forks, and in doing so allows HR-mediated repair of the DNA lesions^41^. Unlike with *BRCA1/2*-deficient cells where olaparib sensitivity is based on their inability to perform HRR, the genotoxic consequence of olaparib treatment in *ATM*-deficient cells is largely due to toxic NHEJ^49^. Based on literature reports showing an increased sensitivity of *ATM*-deficient cells to loss or inhibition of DNA-PK^25–27,52^, we explored the combination efficacy of olaparib and AZD7648 in *ATM*-deficient preclinical models. We found that olaparib single-agent treatment led to an increased number of chromosomal aberrations, as well as growth inhibition in both our FaDu and A549 ATM KO cell models compared to ATM WT cells, while the combination of AZD7648 and olaparib led to greater cell growth inhibition compared to single-agent treatment and apoptosis specifically in ATM KO cells compared to ATM WT. The increase in micronuclei formation and the chromosomal aberration profile we observed following combination treatment suggested that we are achieving an additive efficacy of the two agents in ATM KO cells in vitro, which translates to profound tumour regressions in vivo. Interestingly, the observed additivity of the combination and the different classes of chromosomal aberrations caused by single agent AZD7648 or olaparib treatment imply that the observed DNA damage occurs via distinct mechanisms in ATM KO cells when comparing these two agents. The combination benefit observed in *ATM*-deficient cells is in contrast to previous studies where NU7441 or KU-0060646 treatment were reported to rescue the cytotoxic effects of PARP inhibitor treatment in *ATM*-deficient cell lines due to a proposed role of DNA-PK activity in promoting toxic NHEJ^42,53^. Our findings however show that AZD7648 treatment does not attenuate the number of chromosomal fusions caused by olaparib treatment in ATM KO cells, which is in line with recent literature suggesting that toxic NHEJ of broken replication forks formed upon topotecan or olaparib treatment are mediated by XRCC4 and ligase IV and not by DNA-PKcs^41^.

While the regulation of pathway choice between HRR and NHEJ by ATM is a plausible explanation for olaparib sensitivity in *ATM*-deficient cells^39,40^, the mechanistic underpinnings of the ATM and DNA-PKcs synthetic lethal interaction is unclear^52^. Our metaphase spread analysis data however do suggest that AZD7648 promotes DNA damage formation through a process independent of replication, as mainly chromosome breaks were detected and they generally form independently of cell cycle phase^54^. Nonetheless, the profound tumour regressions achieved in FaDu ATM KO xenografts show a clear combination benefit for DNA-PK and PARP inhibition in the *ATM*-null genetic background. Furthermore, the combination benefit observed in olaparib-sensitive HR-proficient, as well as in *BRCA1* and *BRCA2*-deficient PDX models, suggest that there are likely other genetic vulnerabilities that sensitize to the combination. We are hopeful that future preclinical and clinical studies will elucidate what these might be.

The magnitude of the AZD7648 and olaparib combination treatment response in the FaDu ATM KO xenografts in vivo (where tumour volumes started to regress after 10 days of treatment) was more striking than the combination in vitro, suggesting that the length of treatment and the accumulation of DNA damage is an important factor in achieving significant anti-tumour effects. The continuous dosing of the combination treatment of up to 70 days was well tolerated in mice. Nonetheless, we demonstrated that AZD7648 administered intermittently alongside continuous olaparib dosing can also achieve tumour regression or tumour growth inhibition (stable disease) that significantly improves upon olaparib dosed on its own. What appears to discriminate regressions from stable disease using these schedules is the total amount of drug delivered in a 21-day period, although future preclinical studies may help elucidate this further. The ability to dose in an intermittent or continuous manner allows various schedules of AZD7648 in combination with olaparib to achieve anti-tumour efficacy, which provides options for adapting AZD7648 treatment in the clinic depending on the tolerability achieved.

The sustained interest in the development of DNA-PK inhibitors is evident from the recent emergence of DNA-PK inhibitors that have progressed to clinical trials^20–24^. The well-established role of DNA-PK in the DDR makes it an attractive therapeutic target, especially as a combination partner for other DDR-targeted chemo- or radiotherapeutics. Additionally, the proposed involvement of DNA-PKcs in the RSR^13–15^ also suggests that DNA-PK inhibitors could be useful in targeting tumours with high levels of replication stress, either as a single agent or in combination with other agents that modulate the RSR, such as ATR inhibitor AZD6738. Besides its role in maintaining genomic stability, DNA-PK has been implicated in a plethora of other cellular processes important for cancer, such as hormone-driven transcription, hypoxia and the inflammatory response^11^. It is therefore not unexpected that overexpression of DNA-PKcs has been found to be associated with advanced tumour stage and poor overall survival in various solid and haematological cancer types^55^. Altogether, these observations underscore the need for the clinical evaluation of DNA-PK inhibitors such as AZD7648 for cancer therapy.

In summary, we have identified AZD7648, a potent and selective inhibitor of DNA-PK that has broad potential for development as an anticancer agent, acting as a sensitizer to a range of DNA DSB-inducing agents, including radiation, cytotoxic chemotherapy and the PARP inhibitor, olaparib. Through its differentiated mechanism-of-action as an inhibitor of NHEJ, AZD7648 has potential to be combined with other DDR-targeted agents to treat a broader range of tumour types and achieve deeper and more sustained responses to current therapies.

## Methods

### Chemistry

Detailed synthesis protocols and characterization of compounds can be found in the Supplementary Materials and Methods section of the Supplementary Information.

### Cell lines

All cell lines tested negative for *Mycoplasma* and were authenticated by short tandem repeat analysis. A full list of cell lines, their origins, cell growth media and assay media used in this study can be found in Supplementary Table 4. The FaDu cell line with all three copies of *ATM* knocked out was generated using transcription activator-like effector nucleases in-house (Discovery Sciences, AstraZeneca, Sweden). The A549 cell line with *ATM* knocked out was generated by CRISPR-Cas9 in-house (Oncology R&D, AstraZeneca, UK).

### Cellular pharmacology

Primary potency of AZD7648 on DNA-PK activity was evaluated in A549 cells following IR by measuring DNA-PKcs autophosphorylation on Ser2056. The selectivity of AZD7648 was evaluated in other cell assays for its pharmacological activity on ATM (pATM Ser1981) in HT29, ATR (pCHK1 Ser345) in HT29, mTOR (pAKT Ser473) in MDA-MB-468, and PI3K isoforms (pAKT Thr308) in BT474 (PI3Kα), MDA-MB-468 (PI3Kβ), RAW-264 (PI3Kγ) and JEKO-1 (PI3Kδ) cells. IC_50_ was defined as the concentration required to give a 50% reduction in the phosphorylation of the downstream target for each respective kinase.

### High-content immunofluorescence imaging and analysis

High-content imaging assays were performed in A549 and OAW42 cells to evaluate the cellular response to combination treatment of AZD7648 with IR and doxorubicin, respectively. Cells were fixed and stained for γH2AX, 53BP1 and pATM Ser1981 and imaged on the CV7000 high-content imaging platform (Yokogawa). Analysis was performed on a Columbus™ image data storage and analysis system (Perkin Elmer).

### Doxorubicin combination synergy score analysis

A synergy score was determined for the effect of compound combination treatments using data generated from the Live/Dead and CellTiter-Glo assays in Genedata Screener software. The synergy score reflects the excess in which the observed effect was greater than the predicted additive effect based on the Loewe additivity model^37^. A score of >0 is indicative of an additive combination effect and ≥5 is indicative of synergistic combination activity.

### In vivo studies

Immunocompromised SCID (C.B-17/IcrHan®Hsd-*Prkdc*^*scid*^) or Hsd:Athymic Nude-*Foxn1*^*nu*^ female mice (Envigo) were used for tumour implantation. AZD7648 was formulated in 0.5% hydroxypropyl methylcellulose/0.1% Tween80 (HPMC/T) and orally dosed (4–100 mg kg^−1^). When dosed twice daily, the time between the morning and evening doses was 8 h. Targeted irradiation of 2 Gy was delivered over 2 min daily over the first 5 days of treatment. Liposomal doxorubicin (Doxoves) was diluted in physiological saline and intravenously dosed at 2.5 or 5 mg kg^−1^ once per week. Olaparib was formulated in 10% DMSO/30% Kleptose and orally dosed at 100 mg kg^−1^ once daily. All three combinations were dosed 1 h after the morning dose of AZD7648 or its vehicle HPMC/T. Tumour growth inhibition from start of treatment was assessed by comparison of the mean change in tumour volume for the control and treated groups, using the Mousetrap application and represented as TGI. Statistical significance was evaluated using a one-tailed *t*-test. All in vivo studies complied with all relevant ethical regulations for animal testing and research, followed AstraZeneca’s global bioethics policy and received ethical approval from the AstraZeneca ethical committee. HBCx-17 PDX study was carried out at XenTech, France in accordance with French regulatory legislation concerning the protection of laboratory animals. CTG-703, CTG-0828 and CTG-0149 PDX studies were carried out at Champions Oncology, Inc., USA in accordance to the guidelines of the Institutional Animal Care and Use Committee (IACUC) of Champions Oncology and the USA regulatory legislation. CDX and OV2022 PDX studies were conducted in the UK in accordance with UK Home Office legislation, the Animal Scientific Procedures Act 1986 and the Home Office project licences 70/8839, 70/8894 and P0EC1FFDF.

### Statistics

Data from independent experiments were reported as mean ± SEM, unless stated otherwise. Student’s *t*‐test analysis was performed to determine statistical significance in replicate comparisons. For analysis of in vivo tissues samples, two-sided *t*-tests were performed on log-transformed data, assuming unequal variance. To assess the statistical significance of tumour growth inhibition, one-tailed, two-sample, *t*-test with unequal variances was used. To assess the statistical significance of tumour regressions, one-sample *t*-test was used. Table with relevant statistical results for in vivo efficacy studies is provided in Supplementary Table 3.

### Additional materials and methods

Details of antibodies and reagents used for studies and expanded methodology for xenograft studies, cellular pharmacology assays, high-content imaging assays, colony formation assays, radiation combination cell panel screen, Live/Dead assay, CellTiter-Glo assay, cell confluency assay, western blotting, metaphase spread analysis, cell cycle analysis and caspase activity assays can be found in Supplementary Materials and Methods in Supplementary Information and Supplementary Table 5. All uncropped blots are shown in Supplementary Figs. 10–12. Gating strategy used for flow cytometry for cell cycle analysis is shown in Supplementary Fig. 13.

### Reporting summary

Further information on research design is available in the Nature Research Reporting Summary linked to this article.

## Supplementary information

Supplementary Information

Reporting Summary

## Data Availability

All the data supporting the findings of this study are available within the article and its supplementary information files and from the corresponding author upon reasonable request. A reporting summary for this article is available as a Supplementary Information file.
