# Supplementary material for: AZD7648 is a potent and selective DNA-PK inhibitor that enhances radiation, chemotherapy and olaparib activity
Source: Nat Commun. 2019 Nov 7;10:5065. doi: 10.1038/s41467-019-12836-9 (PMC6838110; doi:10.1038/s41467-019-12836-9)
Supplement: Supplementary file 1 — Supplementary Information [file 41467_2019_12836_MOESM1_ESM.pdf]

Supplementary Information

AZD7648 is a potent and selective DNA-PK inhibitor that enhances radiation, chemotherapy and olaparib activity

Fok et al.

Supplementary Table 1. Selectivity of published and clinical DNA-PK inhibitors

Compound IC_{50}	KU-0060648	NU7441	M3814	M9831/VX-984
DNA-PKcs, nM	55	185	43	115
ATM, μM	>30	>3.1	>30	>30
ATR, μM	>30	>30	>30	>30
PI3Kα, μM	0.2	7.8	0.8	>20
PI3Kβ, μM	–	–	0.17	>30
PI3Kγ, μM	–	–	1.59	7.1
PI3Kδ, μM	–	–	0.35	>30
mTOR, μM	0.15	1.8	0.55	>30

Results are presented as geometric mean IC_{50}. DNA-PK(cs), DNA-dependent protein kinase (catalytic subunit); IC_{50}, 50% inhibitory concentration

Supplementary Table 2. Clonogenic survival of A549 and NCI-H1299 NSCLC cells following AZD7648 and IR treatment

AZD7648 (μM)	DEF_{37}		
	n1	n2	Mean
A549			
0.025	1.3	1.2	1.2
0.1	1.4	1.9	1.7
1.21	3.2	7.0	5.1
NCI-H1299			
0.025	1.2	1.3	1.3
0.1	2.9	2,1	2.5
1.21	7.9	7.0	7.4

Related to Figure 1E. Radiosensitivity of A549 and NCI-H1299 cells ± AZD7648 at the indicated concentrations was measured by DEF_{37}, which is the ratio of the radiation dose at survival fraction 0.37 of DMSO-treated cells to the AZD7648-treated cells in this instance. Data presented here are from two independent experiments (n1 and n2). DEF, dose enhancement factor; DMSO, dimethyl sulfoxide; IR, ionizing radiation; NSCLC, non-small-cell lung cancer

Supplementary Table 3 – Statistical analysis for *in vivo* efficacy studies.

A.

	p-value vs.	Day	IR	AZD7648 100 mg/kg	IR + AZD7648 100 mg/kg	IR + AZD7648 50 mg/kg
**A549**	Vehicle	28	2E-02	NS	8E-05	-
	IR	28	-	NS	7E-04	-
**H1299**	Vehicle	17	3E-02	NS	8E-09	7E-07
	IR	17	-	NS	3E-05	7E-04
	Regression	17	NS	NS	2E-04	1E-02
		38	-	-	7E-04	NS

B.

	p-value vs.	Day	Lipo dox 2.5 mg/kg	AZD7648 37.5 mg/kg	Lipo dox + AZD7648 37.5 mg/kg	Lipo dox + AZD7648 24 mg/kg	Lipo dox + AZD7648 12 mg/kg	Lipo dox + AZD7648 4 mg/kg
**BT474**	Vehicle	24	1E-02	NS	7E-07	2E-03	1E-03	4E-03
	Lipo dox	24	-	NS	2E-05	2E-02	NS	NS
	Regression	24	NS	NS	2E-03	-	-	-
	Vehicle	29	4E-03	-	-	4E-04	5E-03	6E-03
	Lipo dox	29	-	-	-	3E-03	5E-02	NS
	Regression	29	NS	-	-	NS	NS	NS
**HBCx-17**	Vehicle	38	6E-08	NS	2E-11	-	--	-
	Lipo dox	38	-	NS	9E-08	-	-	-
	Regression	70	-	NS	NS	-	-	-

C.

	p-value vs.	Day	Olaparib 100 mg/kg	AZD7648 75 mg/kg	Olaparib + AZD7648 75 mg/kg	AZD7648 37.5 mg/kg	Olaparib + AZD7648 37.5 mg/kg
**FaDu ATM KO**	vehicle	18	3E-03	4E-05	2E-11	2E-03	7E-09
	Olaparib	18	-	4E-02	9E-11	NS	2E-07
	Regression	18	NS	NS	3E-03	NS	NS
		53	-	-	1E-06	-	7E-06
**FaDu WT**	Vehicle	14	NS	4E-02	1E-04	1E-02	NS
	Olaparib	14	-	NS	1E-02	NS	NS
**HBCx-17**	Vehicle	38	3E-06	3E-06	8E-05	-	-
	Olaparib	38	-	NS	3E-02	-	-
	Regression	70	NS	-	4E-03	-	-
**CTG-703**	Vehicle	52	9E-04	4E-03	6E-06	-	-
	Olaparib	52	-	NS	8E-03	-	-
	Regression	52	NS	NS	1E-02	-	-
**OV2022**	Vehicle	31	2E-02	2E-03	1E-05	-	-
	Olaparib	31	-	NS	2E-04	-	-
	Regression	31	NS	NS	1E-02	-	-
**CTG-0828**	Vehicle	40	NS	NS	6E-04	-	-
	Olaparib	40	-	NS	4E-03	-	-
	Regression	84	NS	NS	4E-02	-	-
**CTG-0149**	Vehicle	35	NS	3E-03	7E-03	-	-
	Olaparib	35	-	3E-02	8E-03	-	-

D.

	p-value vs.	Day	Olaparib 100 mg/kg	AZD7648 75 mg/kg	Continuous	7ON 21 OFF	7ON 14 OFF	7ON 7 OFF	14ON 14 OFF
**FaDu ATM KO**	Vehicle	18	3E-03	4E-05	1E-06	7E-06	2E-05	3E-03	1E-05
	Olaparib	18	-	4E-02	5E-06	6E-05	4E-04	1E-02	1E-05
	Regression	43	-	-	2E-03	NS	NS	NS (7E-02)	2E-02

To assess statistical significance of tumour growth inhibition, one tailed, two sample, t-test with unequal variances was used. To assess the statistical significance of tumour regressions, one sample t-test was used. P-values are displayed for each treatment group (in columns) in comparison with control treatments or regression. **A.** Studies combining with ionising radiation (IR). **B.** Studies combining with liposomal doxorubicin. **C.** Studies combining with olaparib. **D.** Scheduling study in combination with olaparib.

Supplementary Table 4. Cell lines, DDR-related mutations and assay media

Cell line	Origin	DDR/RS-related mutations (VAF)	*In vitro* assay medium
A549	ATCC	*ATR* splice (0.41)	DMEM + 10% FBS
A549 WT	ATCC	*ATR* mut (0.28)	Ham's F12 + 10% FBS
A549 ATM KO	ATCC	*ATM*del	Ham's F12 + 10% FBS
BT-474	ATCC	*TP53* E285K (1.00), *BRCA2* S3094 (0.35), RAD51C amp	DMEM + 10% FBS
DLD1	ATTC	*N.A.*	RPMI 1640 + 10% FBS
DLD1 BRCA2 (-/-) KO	Horizon	*BRCA2del*	RPMI 1640 + 10% FBS
FaDu	ATCC	*TP53* R428L (0.35)	RPMI 1640 + 10% FBS
FaDu ATM (-/-/-) KO	ATCC	*ATM*del, *TP53* R428L (0.51)	RPMI 1640 + 10% FBS
HCC70		*TP53* R248Q	RPMI 1640 + 10% FBS
HCC1806	ATCC	*TP53* T256fs (0.97), *CCNE1* amp	RPMI 1640 + 10% FBS
HCC1937	ATCC	*BRCA1* Q1756fs (0.94), *FAM35A**	RPMI 1640 + 10% FBS
HT-29		*TP53* R273H (0.96), *MYC* amp	McCoy's 5A + 10% FBS
JEKO-1	ATCC	*MYC* amp	RPMI 1640 + 10% FBS
MDA-MB-231	ATCC	*TP53* R280K (1.00)	DMEM + 10% FBS
MDA-MB-436	ATCC	*BRCA1* splice, *TP53* amp, *RAD51B* del	DMEM + 10% FBS
MDA-MB-468	ATCC	*TP53* R273H (0.93), *FANCD2* splice (0.28), *BLM* D554V (1.00)	DMEM + 10% FBS
NCI-H1299	ATCC	*TP53* del	RPMI 1640 + 10% FBS
OAW42	ECACC	*MSH5*del	DMEM + 10% FBS + 1 mM NaPyr
RAW-264	ECACC	**	DMEM + 1% FBS
SKOV3	ATCC	*TP53* P89fs (0.92), *ATM* splice (0.40)	McCoy's 5A + 10% FBS
SUM149PT	Asterand Bioscience	*BRCA1* N723fs (1.00), *TP53* M237I (0.97), *FANCD2* K50N (0.45)	Ham's F12 + 5% FBS + 10 mM HEPES + 1 μM hydrocortisone + 5 μM insulin
TOV21G	ATCC	*NBN* R446fs (0.46)	Medium 199 + Medium 105 (1:1) + 15% FBS
UWB1.289 + BRCA1	ATTC	*TP53* mutated, *BRCA1* 2594delC (1.00), *BRCA1 complemented*	Medium 171 + MEGS (Lonza) + 3% FBS
UWB1.289	ATCC	*TP53* mutated, *BRCA1* 2594delC (1.00)	Medium 171 + MEGS (Lonza) + 3% FBS

ATCC, American Tissue Culture Collection; AZ, AstraZeneca; DMEM, Dulbecco's modified Eagle's medium; DSMZ, German Collection of Microorganisms and Cell Cultures; ECACC, European Collection of Cell Cultures; DDR/RS (DNA damage response/replication stress)-related mutations generally based on AZ cBioPortal genomic data; VAF, variant allele frequency; N.A., not attributed; *not expressed, **mouse macrophages; FBS, foetal bovine serum; HEPES, 4-(2-hydroxyethyl)-1-piperazineethanesulfonic acid; KO, knockout; MEGS, mammary epithelial growth supplement; MEM, minimum essential medium; NaPyr, sodium pyruvate; NIH, National Institutes of Health; RPMI 1640, Roswell Park Memorial Institute medium; WT, wild type.

Supplementary Table 5. Antibodies and the assays in which they were used

Target	Manufacturer	Catalogue number	Species	Application	Antibody dilution
53BP1	Novus	NB100-304	Rabbit	IF	1:1000
Actin	Cell Signalling	4970	Rabbit	WB	1:5000
pAKT Ser473	Cell Signalling	4060	Rabbit	IF	1:200
pAKT Thr308	Cell Signalling	7144	Mouse	ELISA	1:250
pATM Ser1981	Abcam	Ab81292	Rabbit	WB	1:1000
pATM Ser1981	Millipore	MAB3806	Mouse	IF	1:10000
pCHK1 Ser345	Cell Signalling	2348	Rabbit	IF	1:100
pCHK2 Thr68	Cell Signalling	2661	Rabbit	WB	1:1000
CHK2	Millipore	05-649	Mouse	WB	1:1000
pDNA-PKcs Ser2056	AstraZeneca		Rabbit	WB, ELISA, IHC	1:500, 1:6500, 1:425
DNA-PKcs	Cell Signalling	12311	Mouse	WB	1:1000
DNA-PKcs	Abcam	1832	Rabbit	WB, ELISA	1:1000, 1:400
GAPDH	Cell Signalling	2118	Rabbit	WB	1:5000
γH2AX	Cell Signalling	9718	Rabbit	WB, IHC	1:1000, 1:1000
γH2AX	Cell Signalling	2577	Rabbit	WB	1:1000
γH2AX	Millipore	05-636	Mouse	IF, WB	1:1000
H2AX	Abcam	ab20669	Rabbit	WB	1:1000
PAR	Trevigen	4336	Rabbit	WB	1:500
PARP1 cleaved	Cell Signalling	5625	Rabbit	WB	1:1000
pRPA32 Ser4/Ser8	Bethyl Laboratories	A300-245-A	Rabbit	WB	1:1000
RPA32	Bethyl Laboratories	A300-244-A	Rabbit	WB	1:2000
Vinculin	Sigma	19191	Mouse	WB	1:5000
Anti-rabbit IgG, HRP conjugated	Cell Signalling	7074	Goat	WB	1:1000
Anti-mouse IgG, HRP-conjugated	Cell Signalling	7076	Horse	WB	1:1000
Alexa Fluor® 488 anti-rabbit IgG	ThermoFisher	A-11008	Goat	IF	1:1000
Alexa Fluor® 647 anti-mouse IgG	ThermoFisher	A-21235	Goat	IF	1:1000

DNA-PKcs, DNA-PK catalytic subunit; ELISA, enzyme-linked immunoabsorbant assay; HRP, horseradish peroxidase; IF, immunofluorescence; IgG, immunoglobulin G; IHC, immunohistochemistry; WB, western blotting

Supplementary Figure 1. AZD7648 treatment leads to the persistence of DNA DSBs following 2 Gy IR as measured by the neutral comet assay.

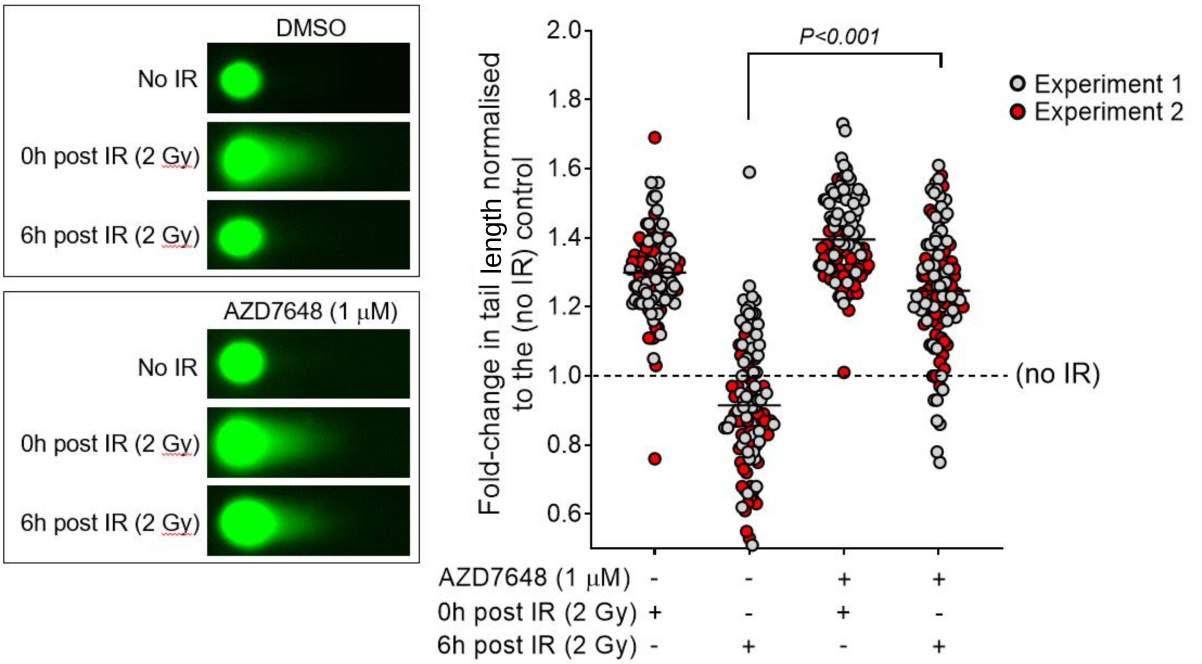

Related to Figure 1. Neutral comet analysis of untreated (No IR) or IR (2 Gy)-treated A549 cells in absence (DMSO) or presence of 1 μM AZD7648 that were either immediately processed (0h post IR) or left to recover from radiation treatment for six hours (6h post IR). Per experiment, tail lengths from 50 cells are plotted. Graphs represent mean tail length ± SD (n=2); one-way ANOVA with Tukey's multiple comparisons test where $P \leq 0.05$ is significant.

Supplementary Figure 2. Bodyweights of mice in xenograft studies. AZD7648 combinations with IR, liposomal doxorubicin and olaparib are tolerated *in vivo*, and experimental mouse body weights are maintained within an acceptable range.

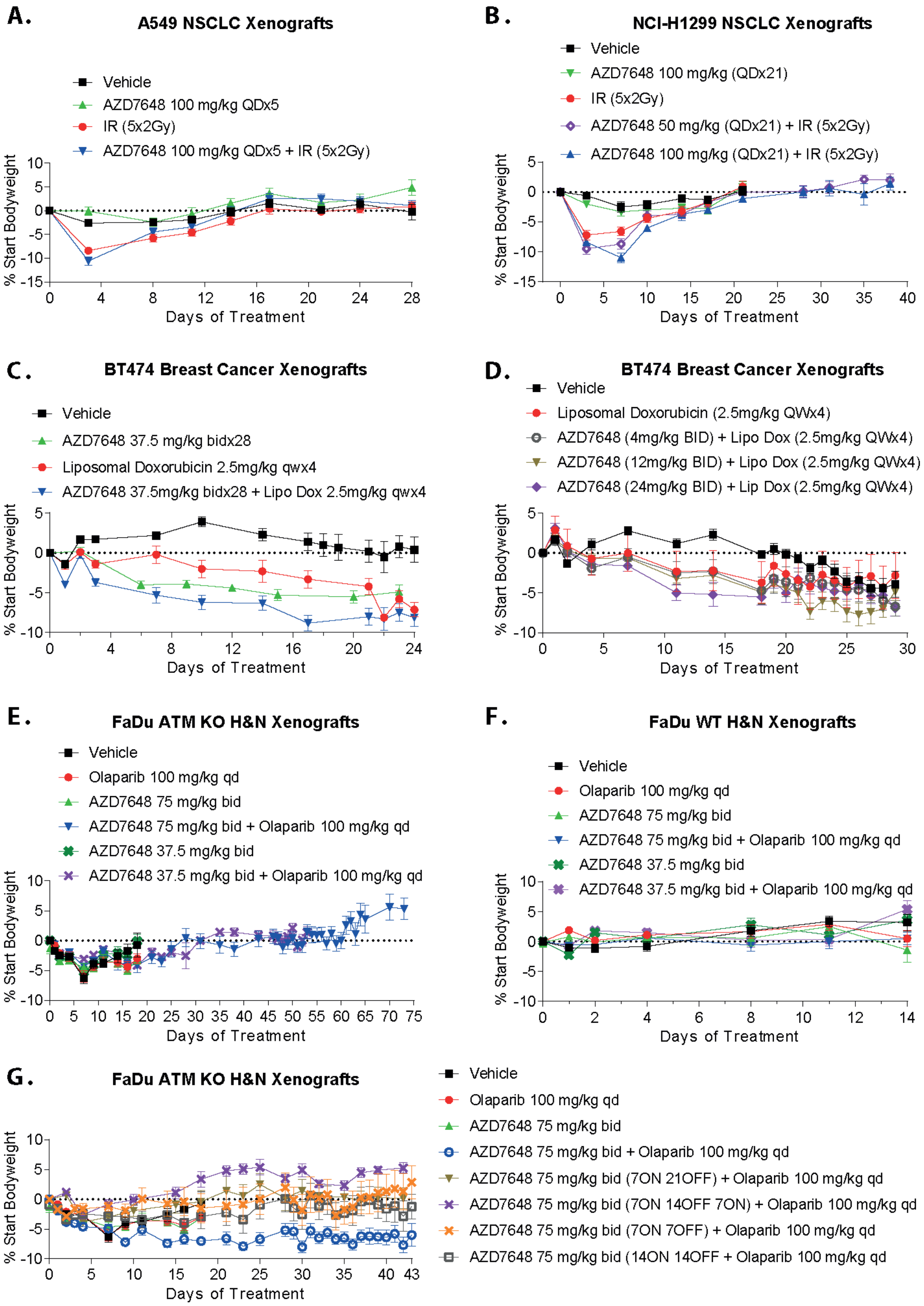

A) Related to Figure 2A. AZD7648 + IR combination in A549 xenografts. **B**) Related to Figure 2B. AZD7648 + IR combination in NCI-H1299 xenografts. **C**) Related to Figure 4A. AZD7648 + liposomal doxorubicin combination in BT-474 xenografts. **D**) Related to Supplementary Figure 4. AZD7648 + liposomal doxorubicin combination dose response in BT-474 xenografts. Relative to Figure 4A. **E**) Related to Figure 5C. AZD7648 + olaparib combination in FaDu ATM knockout (KO) xenografts. **F**) Related to Supplementary Figure 8. AZD7648 + olaparib combination in FaDu WT xenografts. **G**) Related to Figure 8. Scheduling of AZD7648 + olaparib combination in FaDu ATM KO xenografts.

Supplementary Figure 3. Pharmacodynamic modulation of DNA-PKcs biomarkers after dosing of AZD7648 and IR in NCI-H1299 xenografts. Related to Figure 2C. Measured by Western blot (0h n=8, other treatments n=7). Several comparisons between IR and IR + AZD7648 were found to be statistically significant using a one-way ANOVA. For pDNAPK-cs Ser2056: 3h ($p=0.01$). For γH2AX: 3h ($p<0.0001$). For pRPA32 Ser4/Ser8: 0.5h ($p<0.0001$), 3h ($p<0.0001$) and 7h ($p=0.0002$).

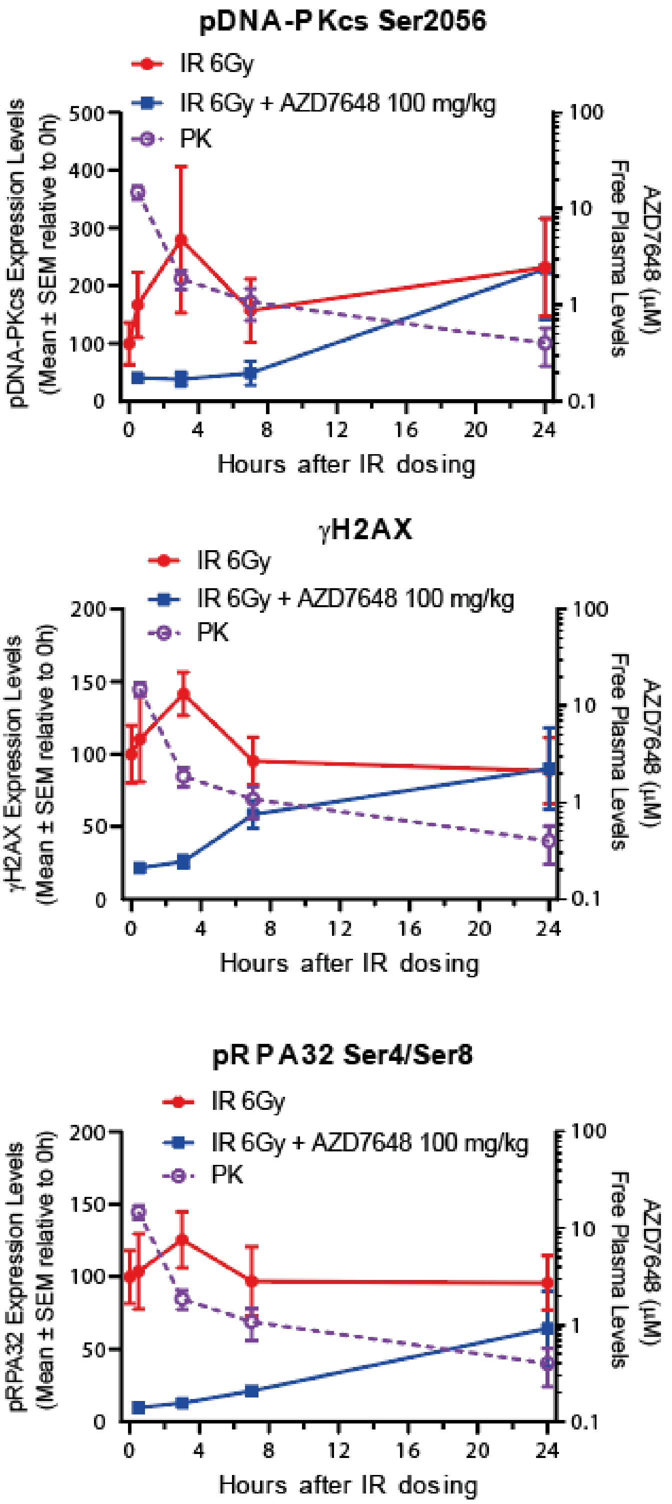

Supplementary Figure 4. Synergistic dose-dependent tumour growth inhibition was clearly observed *in vivo* in BT474 breast xenografts treated with different doses of AZD7648 and liposomal doxorubicin. Related to Figure 4A. Vehicle n=11, liposomal doxorubicin n=10, 4 mg/kg combination n=11, 12 and 24 mg/kg combinations n=9. Corresponding mouse bodyweights and statistical analysis can be found in Supplementary Figure 2D and Supplementary Table 3B. To assess tumour growth inhibition, one tailed, two sample, t-test with unequal variances was used and for tumour regression, one sample t-test analysis.

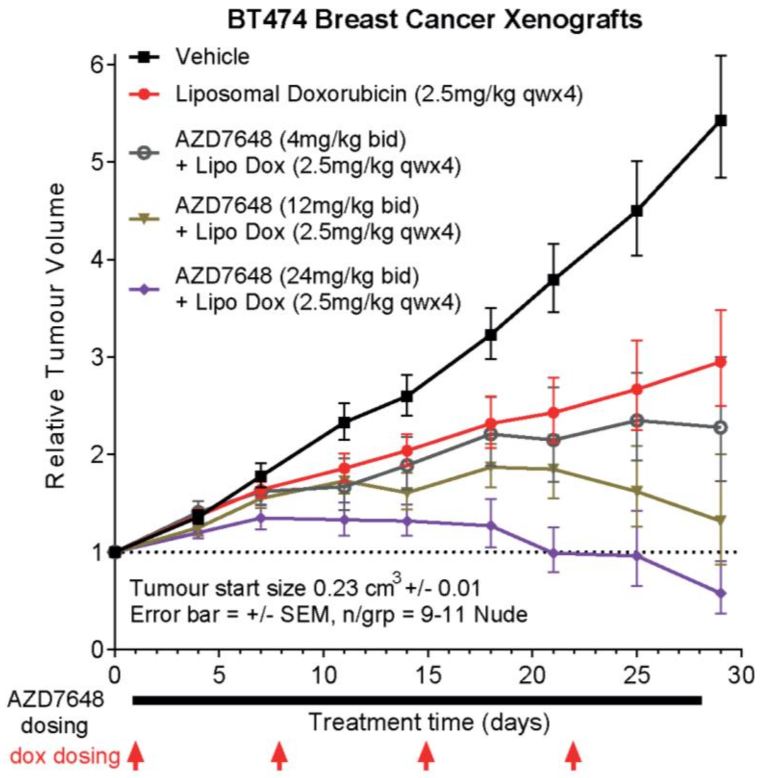

Supplementary Figure 5. Bodyweights of mice in PDX studies.

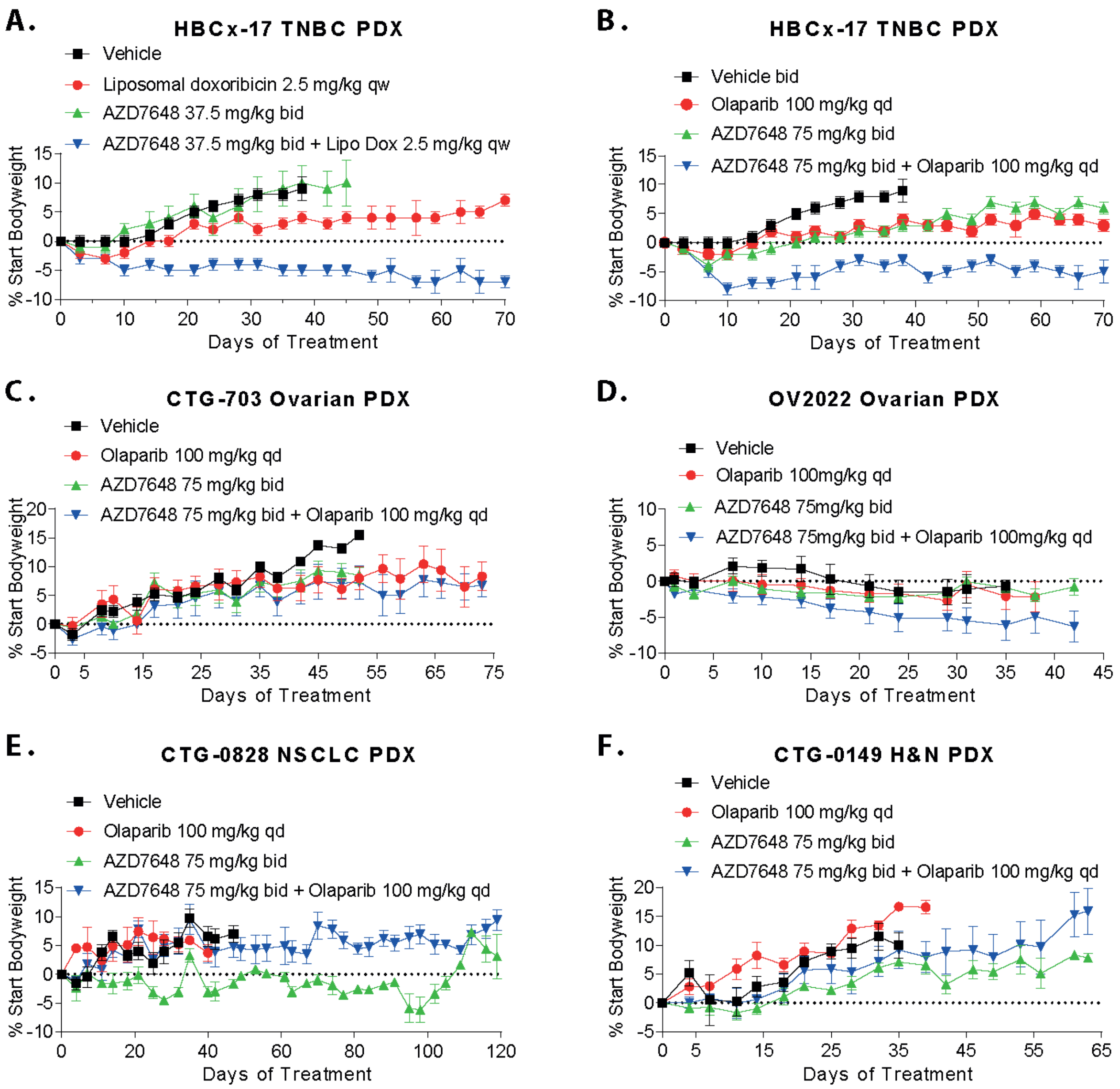

A) Related to Figure 4B. AZD7648 + liposomal doxorubicin combination in HBCx-17 patient-derived xenograft (PDX). **B**) Related to Figure 6A. AZD7648 + olaparib combination in HBCx-17 PDX. **C**) Related to Figure 6B. AZD7648 + olaparib combination in CTG-703 PDX. **D**) Related to Figure 6C. AZD7648 + olaparib combination in OV2022 PDX. **E**) Related to Figure 6D. AZD7648 + olaparib combination in CTG-0828 PDX. **F**) Related to Figure 6E. AZD7648 + olaparib combination in CTG-0149 PDX.

Supplementary Figure 6. ATM KO cells showed greater sensitivity to AZD7648 and olaparib monotherapy compared with their respective WT cells. Deficiency in BRCA1/2 did not modulate cellular sensitivity to AZD7648 monotherapy.

A

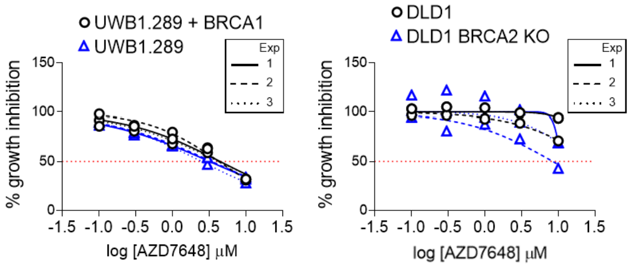

**AZD7648**		**µM**			
**Cell line**	**pGI_{50} ± SEM**	**GI_{50}, n**	**95% confidence interval**		
UWB1.289 + BRCA1	5.362 ± 0.012	4.324, 3	4.219	to	4.485
UWB1.289	5.519 ± 0.073	3.025, 3	2.557	to	3.658
DLD1	4.496 ± 0.0002	31.950, 3	31.933	to	31.970
DLD1 BRCA2-/-	4.907 ± 0.198	12.398, 3	7.866	to	20.748

B

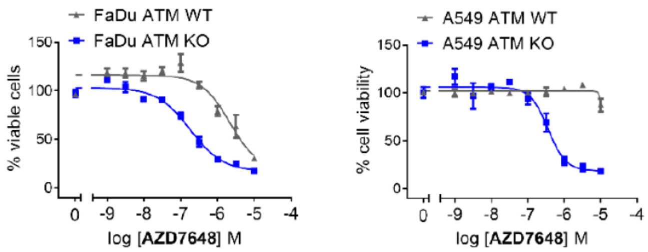

**AZD7648**		**µM**			
**Cell line**	**pGI_{50} ± SEM**	**GI_{50}, n**	**95% confidence interval**		
FaDu ATM KO	6.258 ± 0.112	0.536, 4	0.429	to	0.710
FaDu WT	5.270 ± 0.174	5.368, 3	3.410	to	8.451
A549 ATM KO	6.284 ± 0.013	0.520, 3	0.502	to	0.538
A549 WT	5.160 ± 0.237	6.915, 3	3.726	to	12.833

C

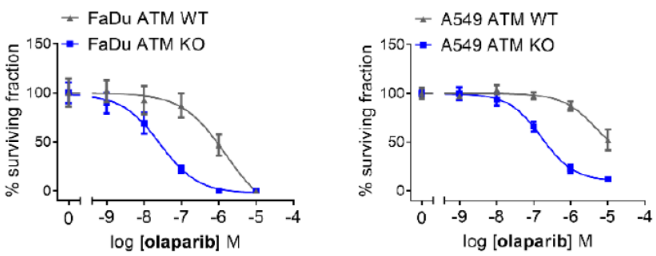

**Olaparib**		**µM**			
**Cell line**	**pIC_{50} ± SEM**	**IC_{50}, n**	**95% confidence interval**		
FaDu ATM KO	7.846 ± 0.245	0.014, 3	0.008	to	0.027
FaDu WT	6.198 ± 0.429	0.633, 3	0.207	to	1.935
A549 ATM KO	6.601 ± 0.167	0.251, 3	0.162	to	0.387
A549 WT	4.943 ± 0.064	11.392, 3	9.630	to	13.476

Concentration-dependent response to AZD7648 monotherapy treatment was measured by Live/Dead assay in **A**) UWB1.289 and UWB1.289 complemented with wild-type *BRCA1* (UWB1.289 + BRCA1), DLD1 and DLD1 with the *BRCA2* gene knocked out (DLD1 BRCA2 KO), and **B**) FaDu and A549 cells with the *ATM* gene knocked out (ATM KO) compared with their WT counterpart. Graphs represent percentage viable cells ± SD following 7 days' treatment relative to DMSO vehicle-treated controls from one representative experiment of at least three independent experiments. Tables describe AZD7648 GI_{50} of UWB1.289, UWB1.289 + BRCA1, DLD1, DLD1 BRCA2 KO, and FaDu and A549 ATM KO cells compared with their WT counterparts following 7 days' treatment. Mean pGI_{50} (–log [GI_{50}]) ± SEM, geometric mean GI_{50}, number of experimental repeats (n) and 95% confidence interval are described. **C**) Colony formation assay was performed on FaDu and A549 ATM KO and WT cells treated with increasing concentrations of olaparib. Graphs represent percentage surviving fraction of colonies ± SD following 10–14 days' treatment relative to DMSO vehicle-treated control from one representative experiment of three independent experiments. Table describes AZD7648 IC_{50}. Mean pIC_{50} ± SEM, geometric mean IC_{50}, number of experimental repeats (n) and 95% confidence interval are described. The data presented in this figure support work presented in Figure 5.

Supplementary Figure 7. AZD7648 and olaparib combination leads to increased DNA damage and a greater antiproliferative effect compared with monotherapy *in vitro*

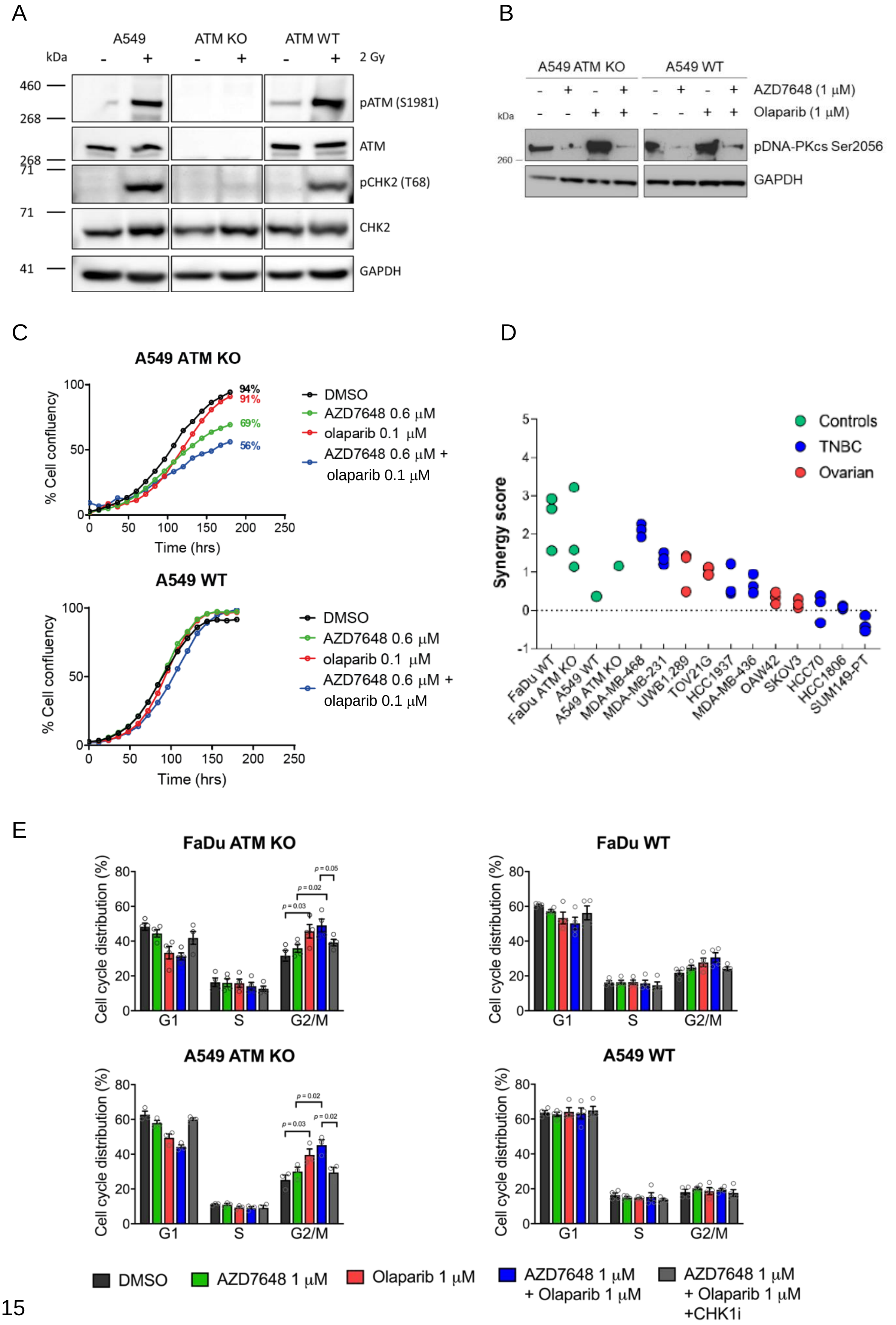

F

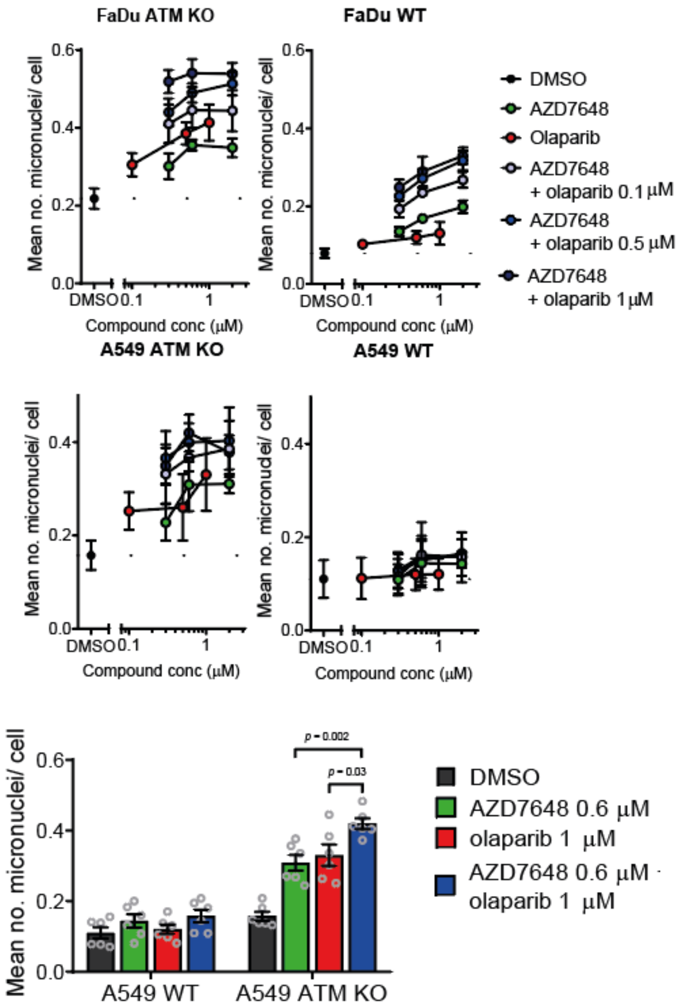

G

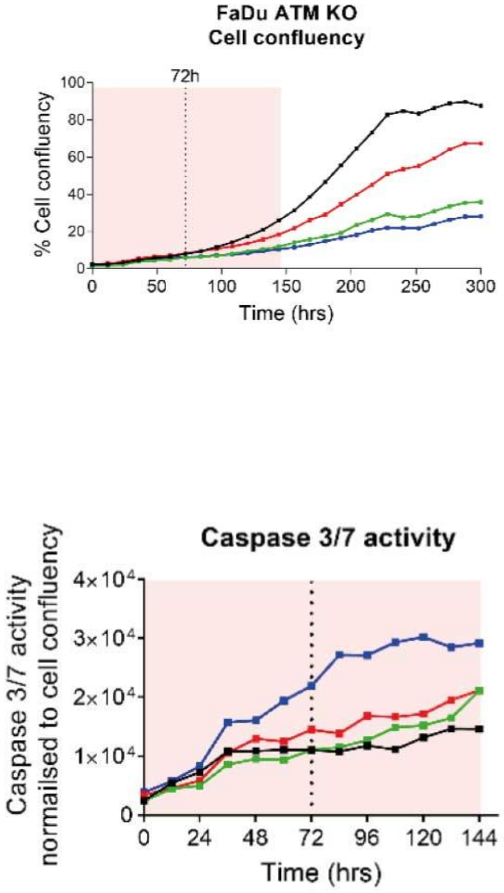

A) Western blot analysis of whole-cell lysates from A549 parental, ATM KO and WT cells ±2 Gy radiation treatment. Membranes were probed with antibodies against phospho-ATM (pATM Ser1981), ATM, phospho-CHK2 (pCHK2 Thr68) and CHK2. **B**) Western blot analysis of whole-cell lysates from A549 ATM KO and WT cells treated with AZD7648, olaparib or its combination at indicated concentrations collected following 24 hours' treatment. Membranes were probed with antibodies against phospho-DNA-PKcs (pSer2056) and γH2AX (phosphorylated on Ser139) with glyceraldehyde 3-phosphate dehydrogenase (GAPDH) as loading control. Related to Figure 5A. **C**) Cell confluency of A549 ATM KO and WT cells was measured every 12 hours following AZD7648, olaparib monotherapy or combination treatment at indicated concentrations with an IncuCyte® system. Graphs represent mean percentage cell confluency from duplicate wells from one representative experiment of three independent experiments. Percentage values indicate final cell confluency at 144-hour time point for each condition. Related to Figure 5B. **D**)

Synergy scores for AZD7648 and olaparib combination in a panel of four ovarian and seven breast cancer cell lines. Viability of cells treated with a combination 5x5 matrix of AZD7648 (0.1 – 10 μM) and olaparib (0.1 – 10 μM) concentrations for 5–7 days was measured by the Live/Dead assay. Synergy scores were determined based on the Loewe model of additivity using Genedata Screener software. Data points represent synergy score determined from each independent experiment. A score of >0 is indicative of combination benefit. The FaDu and A549 ATM KO isogenic pairs were run as controls. **E**) Cell cycle distribution of FaDu ATM KO, FaDu WT, A549 ATM KO and A549 WT cells following 48-hour AZD7648, olaparib monotherapy or combination treatment ± CHK1 inihbitor (LY2606368, 70 nM). Cells were fixed and stained with DAPI, and DNA content was analysed by flow cytometry. Data are shown as mean percentage cell cycle distribution (mean ± SEM (n = 4); unpaired t-test, $P \leq 0.05$ is significant. **F**) Frequency of micronuclei formation in A549 ATM KO and WT cells following 48- and 72-hour AZD7648 and olaparib monotherapy and combination treatment. Graphs represent mean number of micronuclei per cell ± SEM from two independent experiments. *P* values were calculated using an unpaired t-test where $P \leq 0.05$ is significant. Related to Figure 7A. **G**) Cell confluency (top) with accompanying caspase 3/7 activity (bottom) over the time highlighted in the pink box. FaDu ATM KO cells were treated with AZD7648, olaparib or the combination at indicated concentrations. Cell confluency was measured every 12 hours, and caspase 3/7 activity was detected using IncuCyte caspase 3/7 apoptosis reagent on an IncuCyte system. The bottom graph represents mean fluorescence levels normalized for total cell confluency from technical triplicates. Representative data from n=3 experiments are shown. Related to Figure 7C.

Supplementary Figure 8. AZD7648 in combination with olaparib is more efficacious in FaDu ATM KO xenografts than in FaDu WT xenografts. FaDu WT xenograft experiments were stopped after day 14 due to tumour condition (ulceration). On the same day, FaDU ATM KO tumours treated with the AZD7648 75 mg/kg + olaparib combination were already heading tumour regression (SCID mice, vehicle and AZD7648 37.5 mg/kg n=7, olaparib n=5, AZD7648 75 mg/kg n=4, combination 37.5 mg/kg n=8, combination 75 mg/kg n=10, geometric mean ± SEM). Related to Figure 5C. Corresponding mouse bodyweights and statistical analysis can be found in Supplementary Figure 2F and Supplementary Table 3C. To assess tumour growth inhibition, one tailed, two sample, t-test with unequal variances was used and for tumour regression, one sample t-test analysis.

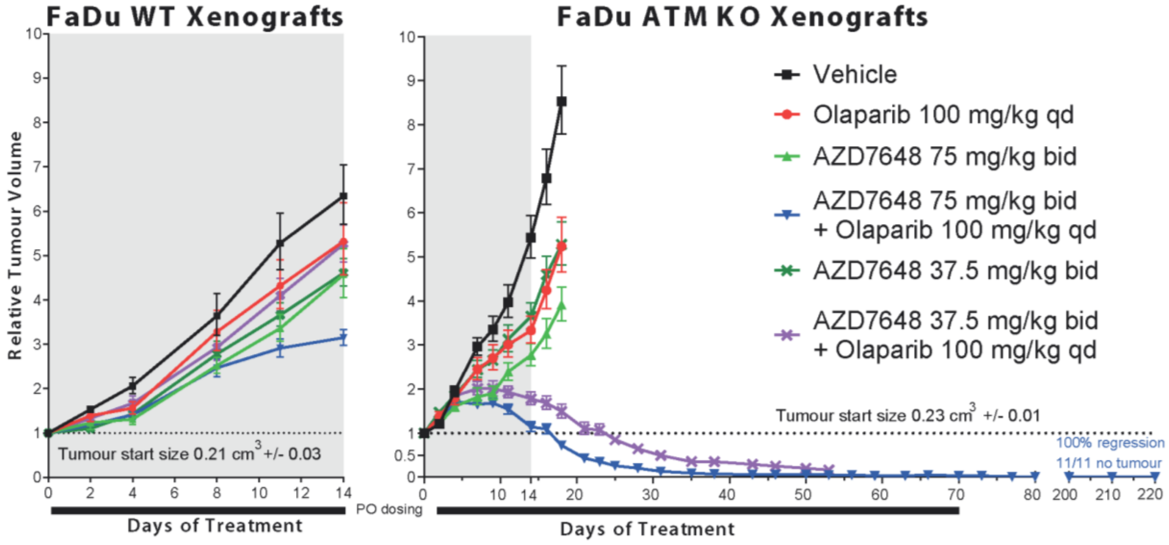

Supplementary Figure 9. AZD7648 75 mg/kg reduced levels of pDNA-PKcs Ser2056, γH2AX and pRPA32 Ser4/Ser8 in FaDU ATM KO xenografts. Related to Figure 5D. Measured by Western blot (0h n=11, 0.5h, 2h and 8h n=4, 24h n=5, 4h n=3). Several comparisons between vehicle and AZD7648 75 mg/kg treatments were found to be statistically significant using a one-way ANOVA. For pDNA-PKcs Ser2056: 2h (p=0.0007) and 4h (p=0.03). For γH2AX: 0.5h (p<0.0001), 2h (p=0.003) and 4h (p=0.01). For pRPA32 Ser4/Ser8: 0.5h (p<0.0001), 2h (p<0.0001), 4h (p=0.0002), 8h (p<0.0001) and 24h (p=0.008). PK, pharmacokinetics of AZD7648

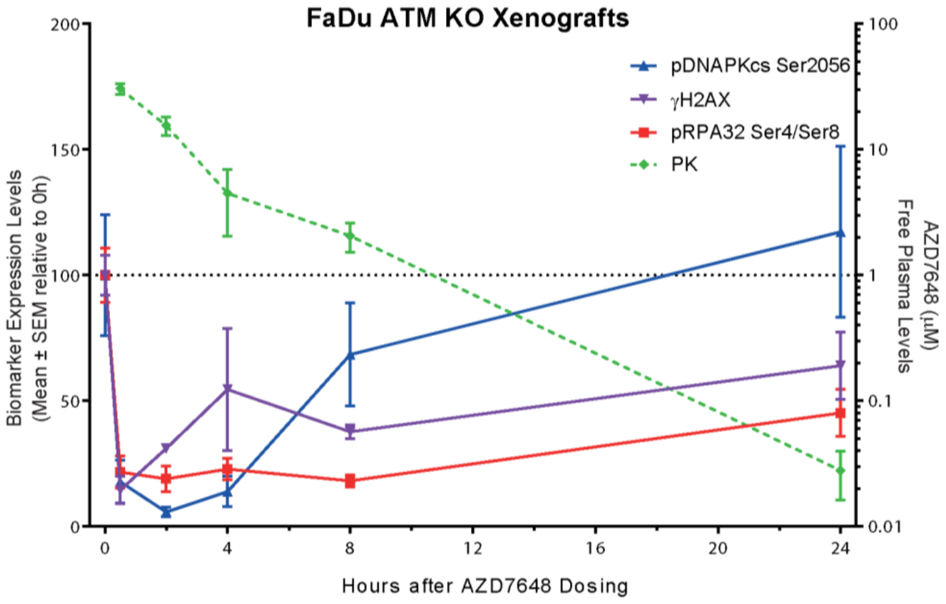

Supplementary Figure 10. Uncropped blot images corresponding to Figure 1B

Size of protein ladder indicated with arrows on scanned image of film.

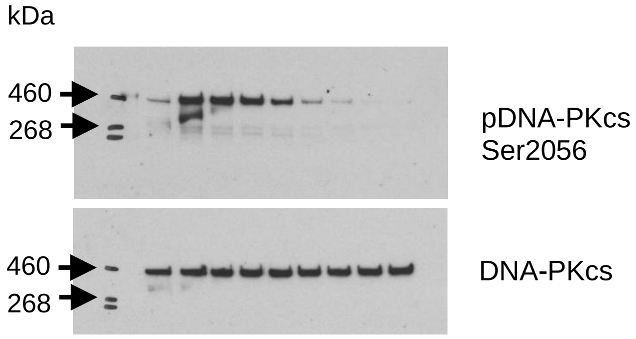

Supplementary Figure 11. Uncropped blot images corresponding to Figure 3A
Size of protein ladder indicated with arrows on image showing blot captured under visible light overlaid with chemiluminescent bands captured at higher exposure than bands presented in final figure. Black box indicates bands that are presented in final figure.

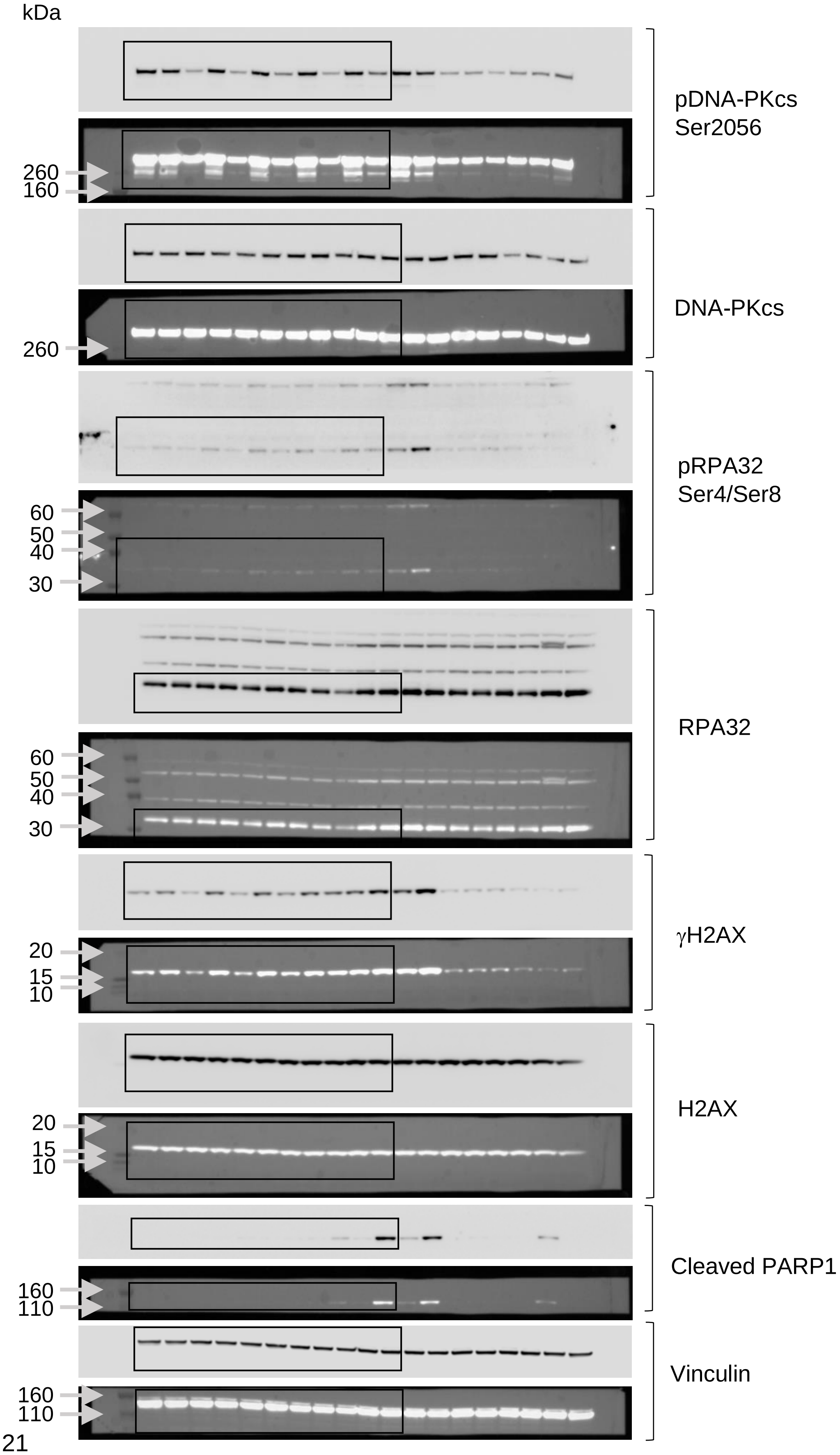

Supplementary Figure 12. Uncropped blot images corresponding to Figure 5A

Size of protein ladder indicated with arrows on image showing blot captured under visible light overlaid with chemiluminescent bands captured at higher exposure than bands presented in final figure. Black box indicates bands that are presented in final figure.

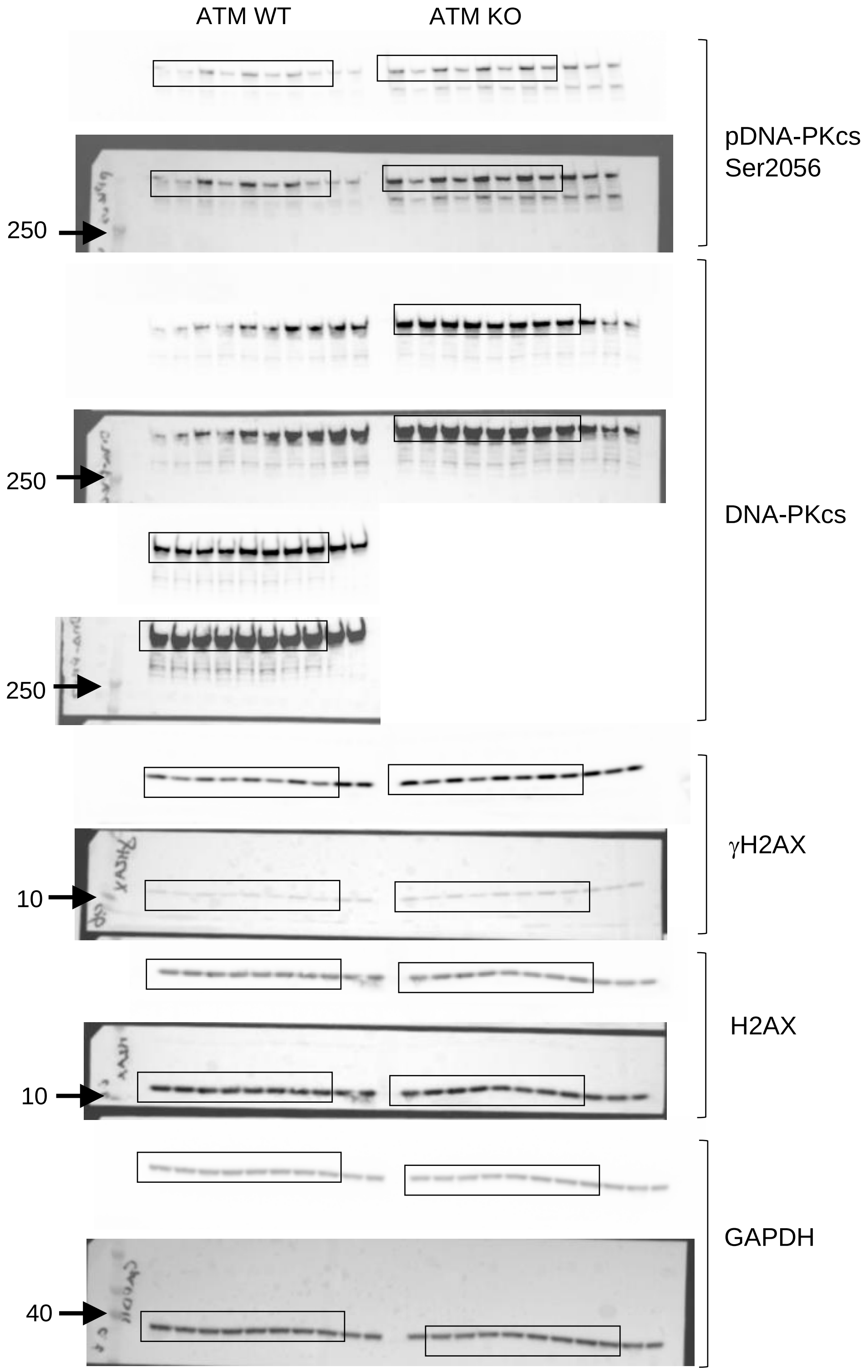

Supplementary Figure 13. Gating strategy for flow cytometry data

A

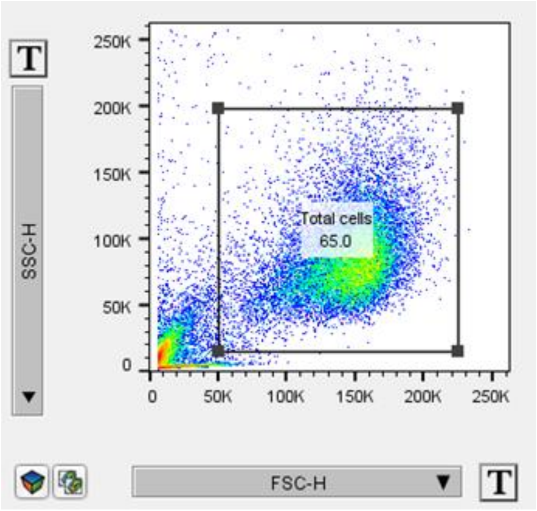

B

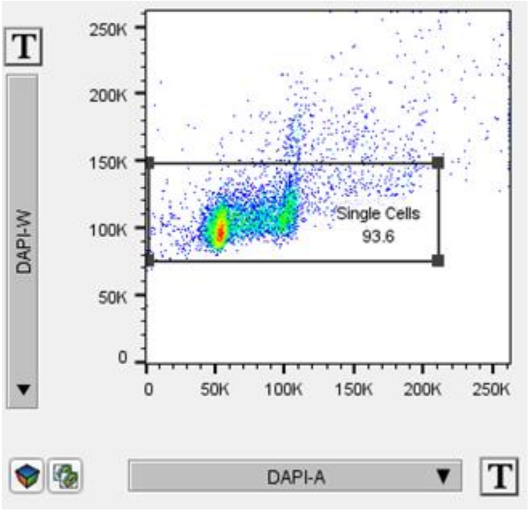

C

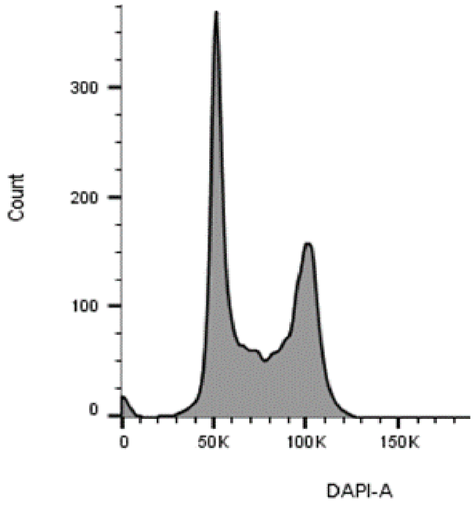

A) Total cell population was gated using FSC-H (x axis) and SSC-H (y axis). **B**) Single-cell populations were gated using DAPI area (x axis) and width (y axis). Doublets (where area and width are disproportional to each other) were excluded, but >4N cells were included. **C**) Cell-cycle distribution was observed by plotting DAPI-A against cell number. Cell-cycle gating was carried out based on the width of the peaks of the cell-cycle profile

Supplementary Materials and Methods

Representative synthetic protocol for AZD7648

(*E*)-*N*,*N*-dimethyl-*N'*-(4-methyl-5-nitropyridin-2-yl)formimidamide.

1,1-Dimethoxy-*N*,*N*-dimethylmethanamine (26.0 mL, 196 mmol) was added to 4-methyl-5-nitropyridin-2-amine (10.0 g, 65.3 mmol) in toluene (100 mL) at room temperature (RT). The reaction mixture was heated at reflux for 2 hours and the reaction mixture was allowed to cool to RT. The reaction mixture was concentrated to afford the title compound (13.5 g, 99%) as a yellow solid; ^{1}H NMR (400 MHz, DMSO-*d₆*) δ 8.88 (s, 1H), 8.69 (s, 1H), 6.84–6.79 (m, 1H), 3.17 (s, 3H), 3.06 (d, *J*=0.6 Hz, 3H), 2.53 (d, *J*=0.7 Hz, 3H); ^{13}C NMR (126 MHz, DMSO-d_6) δ 165.5, 157.8, 146.8, 145.1, 140.2, 119.8, 41.1, 35.1, 20.7; HRMS (m/z): MH^+ calculated for $C_9H_{13}N_4O_2$ 209.1039; found 209.1034.

(*E*)-*N*-hydroxy-*N'*-(4-methyl-5-nitropyridin-2-yl)formimidamide.

Hydroxylamine hydrochloride (9.01 g, 130 mmol) was added to (E)-*N*,*N*-dimethyl-*N'*-(4-methyl-5-nitropyridin-2-yl)formimidamide (13.5 g, 64.8 mmol) in MeOH (100 mL) at RT. The reaction mixture was heated at reflux for 1 hour and then allowed to cool to RT. The reaction mixture was partitioned between EtOAc (200 mL) and water (100 mL). The organic layer was isolated and washed with saturated brine (50 mL), passed through phase-separating filter paper and concentrated to afford the title compound (11.9 g, 94%) as a yellow solid; ^{1}H NMR (400 MHz, DMSO-d_6) δ 10.53 (s, 1H), 10.10 (d, *J*=8.7 Hz, 1H), 8.89 (s, 1H), 7.89 (d, *J*=9.0 Hz, 1H), 7.06 (s, 1H), 2.52 (s, 3H); ^{13}C NMR (126 MHz, DMSO-d_6) δ 155.6, 147.0, 145.6, 139.8, 135.0, 112.4, 21.2; HRMS (m/z): MH^+ calculated for $C_7H_9N_4O_3$ 197.0675; found 197.0670.

7-Methyl-6-nitro-[1,2,4]triazolo[1,5-a]pyridine. 2,2,2-Trifluoroacetic anhydride (10.1 mL, 72.8 mmol) was added to (E)-*N*-hydroxy-*N'*-(4-methyl-5-nitropyridin-2-yl)formimidamide (11.9 g, 60.7 mmol) in THF (100 mL) at 0°C. The reaction mixture was stirred at RT for 18 hours and then concentrated. The resulting crude mixture was purified by flash column chromatography (FCC), eluting with 0–100% EtOAc in heptane, to afford an impure pale-orange solid. This solid was recrystallized from heptane:EtOAc, filtered and dried *in vacuo*, then taken up in EtOAc (100 mL), washed with 0.1 M aqueous HCl (50 mL), water (50 mL) and saturated brine (50 mL). The organic layer was passed through phase-separating filter paper and concentrated *in vacuo* to afford the title compound (3.42 g, 32%); ^{1}H NMR (400 MHz, DMSO-d_6) δ 9.97 (s, 1H), 8.73 (s, 1H),8.01–

7.88 (m, 1H), 2.67 (d, *J*=0.8 Hz, 3H); ^{13}C NMR (126 MHz, DMSO-*d*$_6$) δ 157.6, 151.2, 140.2, 136.1, 129.7, 117.2, 20.5; HRMS (m/z): MH$^+$ calculated for $C_7H_7N_4O_2$ 179.0569; found 179.0565.

7-Methyl-[1,2,4]triazolo[1,5-a]pyridin-6-amine. Pd/C (10%, wet support; 0.409 g, 3.84 mmol) was added to 7-methyl-6-nitro-[1,2,4]triazolo[1,5-a]pyridine (3.42 g, 19.2 mmol) and ammonium formate (6.05 g, 96.0 mmol) in ethanol (150 mL) at RT. The reaction mixture was heated at reflux for 2 hours, then allowed to cool to RT, filtered and concentrated to afford the title compound (2.60 g, 91%) as a pale-brown solid; ^{1}H NMR (400 MHz, DMSO-*d*$_6$) δ 8.10 (s, 1H), 8.09 (s, 1H), 7.47 (s, 1H), 5.00 (s, 2H), 2.26 (s, 3H); ^{13}C NMR (126 MHz, DMSO-*d*$_6$) δ 152.3, 145.5, 137.9, 133.6, 114.8, 110.1, 18.2; HRMS (m/z): MH$^+$ calculated for $C_7H_9N_4$ 149.0827; found 149.0822.

Ethyl-2-chloro-4-((tetrahydro-2H-pyran-4-yl)amino)pyrimidine-5-carboxylate. Potassium carbonate (62.5 g, 452 mmol) was added to ethyl 2,4-dichloropyrimidine-5-carboxylate (40 g, 181 mmol) and tetrahydro-2H-pyran-4-amine hydrochloride (24.9 g, 181 mmol) in acetonitrile (1 L). The reaction mixture was stirred at RT for 16 hours. The precipitate was collected by filtration and washed with THF (750 mL), and the organic layers were removed under reduced pressure. The crude product was purified by FCC, elution gradient 0–2% THF in DCM, to afford the title compound (37.7 g, 73%) as a pale-yellow solid; ^{1}H NMR (400 MHz, DMSO-*d*$_6$) δ 8.64 (s, 1H), 8.33 (d, *J*=7.6 Hz, 1H), 4.33 (q, *J*=7.1 Hz, 2H), 4.20 (dddd, *J*=4.3, 8.4, 10.7, 14.9 Hz, 1H), 3.86 (dt, *J*=3.7, 11.8 Hz, 2H), 3.46 (td, *J*=2.3, 11.6 Hz, 2H), 1.84–1.93 (m, 2H), 1.53–1.65 (m, 2H), 1.32 (t, *J*=7.1 Hz, 3H); ^{13}C NMR (126 MHz, DMSO-*d*$_6$) δ 165.9, 163.1, 160.1, 160.8, 104.5, 66.1, 61.8, 47.1, 32.3, 14.4; HRMS (m/z): MH$^+$ calculated for $C_{12}H_{17}ClN_3O_3$ 286.0958; found 286.0948.

2-Chloro-4-((tetrahydro-2H-pyran-4-yl)amino)pyrimidine-5-carboxylic acid. A solution of LiOH (13.1 g, 547 mmol) in water (800 mL) was added to a stirred solution of ethyl 2-chloro-4-((tetrahydro-2H-pyran-4-yl)amino)pyrimidine-5-carboxylate (78.2 g, 273 mmol) in THF (800 mL). The reaction mixture was stirred at RT for 3 hours. The organic layers were removed under reduced pressure. The reaction mixture was acidified with 2 M aqueous HCl. The precipitate was collected by filtration, washed with water (500 mL) and dried under vacuum to afford the title compound (66.4 g, 92%) as a white solid; ^{1}H NMR (400 MHz, DMSO-*d*$_6$) δ 13.76 (s, 1H), 8.60 (s, 1H), 8.55 (d, *J*=7.6 Hz, 1H), 4.18 (tdt, *J*=4.2, 8.3, 11.7 Hz, 1H), 3.85 (dt, *J*=3.6, 11.7 Hz, 2H), 3.46 (td, *J*=2.2, 11.5 Hz,

2H), 1.94–1.85 (m, 2H), 1.62–1.50 (m, 2H); ^{13}C NMR (126 MHz, DMSO-*d*$_6$) δ 167.9, 163.0, 161.4, 161.0, 104.9, 66.1, 46.9, 32.4; HRMS (m/z): MH$^+$ calculated for $C_{10}H_{13}ClN_3O_3$ 258.0640; found 258.0645.

2-Chloro-9-(tetrahydro-2H-pyran-4-yl)-7,9-dihydro-8H-purin-8-one. Triethylamine (25.4 g, 251 mmol) was added to 2-chloro-4-((tetrahydro-2H-pyran-4-yl)amino)pyrimidine-5-carboxylic acid (64.8 g, 251 mmol) and DPPA (69.2 g, 251 mmol) in DMA (330 mL). The reaction mixture was stirred at RT for 1 hour and then stirred at 120°C for 16 hours. The reaction mixture was poured into ice (2 L), and the precipitate was collected by filtration, washed with water (400 mL) and dried under vacuum to afford the title compound (44.8 g, 70%) as a white solid; ^{1}H NMR (400 MHz, DMSO-*d*$_6$) δ 11.62 (s, 1H), 8.14 (s, 1H), 4.42 (t, *J*=12.3 Hz, 1H), 4.03–3.93 (m, 2H), 3.45 (t, *J*=12.1 Hz, 2H), 2.45 (dd, *J*=4.3, 12.8 Hz, 2H), 1.69 (d, *J*=11.6 Hz, 2H); ^{13}C NMR (126 MHz, DMSO-*d*$_6$) δ 153.0, 152.1, 150.5, 134.5, 121.6, 66.8, 49.6, 29.6; HRMS (m/z): MH$^+$ calculated for $C_{10}H_{12}ClN_4O_2$ 255.0649; found 255.0645.

2-Chloro-7-methyl-9-(tetrahydro-2H-pyran-4-yl)-7,9-dihydro-8H-purin-8-one.
A solution of NaOH (31.0 g, 776 mmol) in water (80 mL) was added to a stirred solution of 2-chloro-9-(tetrahydro-2H-pyran-4-yl)-7,9-dihydro-8H-purin-8-one (39.5 g, 155 mmol) and MeI (48.5 mL, 776 mmol) in THF (720 mL). The reaction mixture was stirred at RT for 16 hours. The organic layer was removed under reduced pressure. The reaction mixture was diluted with water. The precipitate was collected by filtration, washed with water (300 mL) and dried under vacuum to afford the title compound (32.5 g, 69%) as a white solid; ^{1}H NMR (400 MHz, DMSO-*d*$_6$) δ 8.37 (s, 1H), 4.47 (tt, *J*=4.3, 12.2 Hz, 1H), 3.98 (dd, *J*=4.6, 11.5 Hz, 2H), 3.46 (td, *J*=1.8, 12.3 Hz, 2H), 3.37 (s, 3H), 2.48–2.39 (m, 2H), 1.77–1.64 (m, 2H). ^{13}C NMR (126 MHz, DMSO-*d*$_6$) δ 152.0, 150.4, 150.2, 133.8, 122.7, 66.3, 49.6, 29.2, 27.4; HRMS (m/z): MH$^+$ calculated for $C_{11}H_{14}ClN_4O_2$ 269.0805; found 269.0804.

7-Methyl-2-((7-methyl-[1,2,4]triazolo[1,5-a]pyridin-6-yl)amino)-9-(tetrahydro-2H-pyran-4-yl)-7,9-dihydro-8H-purin-8-one (AZD7648). Caesium carbonate (24.3 g, 74.4 mmol) was added to 2-chloro-7-methyl-9-(tetrahydro-2H-pyran-4-yl)-7,9-dihydro-8H-purin-8-one (10.0 g, 37.2 mmol) and 7-methyl-[1,2,4]triazolo[1,5-a]pyridin-6-amine (5.51 g, 37.2 mmol) in 1,4-dioxane (200 mL). Brettphos precat G3 (1.69 g, 1.86 mmol) was added and the resulting suspension was stirred vigorously at 100°C for 1 hour. A

further 1% of catalyst was added and the reaction mixture was stirred for a further 30 minutes. The mixture was cooled to RT and filtered, and the solid was washed with 10% MeOH in DCM (100 mL). The filtrate was taken and the solvent was removed *in vacuo*. The resulting crude product was purified by FCC, eluting with 0–10% MeOH in DCM, then by recrystallization from MeOH and DCM to afford the title compound (7.59 g, 54%) as a cream solid, mp 251°C; ^{1}H NMR (400 MHz, DMSO-d_6) δ 8.65 (s, 1H), 9.11 (s, 1H), 8.37 (s, 1H), 8.08 (s, 1H), 7.70 (s, 1H), 4.42 (tt, J=4.1, 12.0 Hz, 1H), 3.97 (dd, J=4.2, 11.4 Hz, 2H), 3.42 (t, J=11.4 Hz, 2H), 3.31 (s, 3H), 2.58–2.52 (m, 2H), 2.40 (s, 3H), 1.72–1.63 (m, 2H). ^{13}C NMR (125.7 MHz, d_4-acetic acid) δ 155.7, 154.4, 153.2, 152.6, 147.9, 141.3, 130.4, 129.4, 124.0, 118.5, 115.7, 67.8, 51.6, 30.6, 28.0,18.9; IR (2% w/w dispersion in KBr) 3453, 1729 cm^{-1}; HRMS (m/z): MH^{+} calculated for $C_{18}H_{21}N_8O_2$ 381.1787; found 381.1778.

Cell lines

A list of cell lines, their origins, and the media used for cell culture maintenance and *in vitro* assays can be found in Supplementary Table 4. For *in vitro* studies, cells were maintained at 37°C, 90% relative humidity and 5% CO_2. For *in vivo* studies, cells were maintained in 7.5% CO_2, and BT-474 and FaDu ATM KO cells were cultured in DMEM + 10% FBS and MEM + 10% FBS, respectively.

Cellular pharmacology

For all cellular pharmacology assays described below, a concentration–response curve was fitted to a four-parameter non-linear regression model using GraphPad Prism or Genedata Screener® software (Genedata, Inc, Basel, Switzerland). IC_{50} was defined as the concentration required to give a 50% reduction in measured response. Details of antibodies used can be found in Supplementary Table 5.

DNA-PKcs pS2056

Pharmacological activity of AZD7648 on DNA-PK activity was evaluated by measuring the inhibition of radiation-induced DNA-PKcs autophosphorylation (S2056) in A549 cells by ELISA. A549 cells were plated 15,000 cells/well in a total volume of 40 μL cell media and incubated overnight. 384-well ELISA plates (Greiner 781077 all-black high-bind) were coated with 0.5 μg/mL DNA-PKcs antibody (Abcam) in phosphate-buffered saline (PBS) overnight at 4°C. Plates were then washed 3x with PBS containing 0.05% Tween-20 (PBS-T) and blocked with 3% bovine serum albumin (BSA) in PBS for ~2 hours, before a

further 3x wash with PBS-T. Test compounds and reference controls were dosed directly into the cell plates using a Labcyte Echo 555 acoustic dispenser. Cell plates were then incubated for 1 hour at 37°C before receiving a radiation dose of 8 Gy (XRAD 160, Precision X-Ray). Cells were incubated for a further 1 hour before removal of cell media. Lysis buffer (in-house preparation with addition of protease inhibitor cocktail tablets [Roche]), 0.1% Tween-20, and 0.1% NP40 was dispensed at 25 μL/well and plates were incubated at 4°C for 15–20 min. Cell lysates (20 μL/well) were transferred to the DNA-PKcs antibody-coated ELISA plates using a CyBio Felix liquid handling platform, and ELISA plates were incubated at 4°C overnight. The following day, ELISA plates were washed 3x with PBS-T and dispensed with in-house DNA-PKcs pSer2056 antibody (0.5 μg/mL in 3% BSA/PBS) at 20 μL/well. Plates were incubated with antibody for 1.5 hours at RT before 3x wash with PBS-T. Goat anti-rabbit HRP secondary antibody (1:2000 dilution in 3% BSA/PBS) was dispensed at 20 μL/well and plates were incubated at RT for 1 hour before 3x wash with PBS-T. QuantaBlu working substrate solution (Thermo Scientific #15169, prepared according to manufacturer's instructions) was dispensed at 20 μL/well and plates were incubated at RT for 1 hour before a further 20 μL/well dispense with QuantaBlu stop solution provided within the kit. The fluorescence intensity of individual wells was determined using a PerkinElmer EnVision plate reader.

ATM pSer1981

Pharmacological activity of AZD7648 on ATM was evaluated by measuring the inhibition of radiation-induced ATM autophosphorylation (Ser1981) in HT-29 cells by an imaging-based assay. HT-29 cells were dispensed into 384-well Greiner plates (#781090) to give 3500 cells/well in a total volume of 40 μL cell media and incubated overnight. Test compounds and reference controls were dosed directly into the cell plates using a Labcyte Echo 555 acoustic dispenser. The cell plates were then incubated for 1 hour at 37°C before receiving a radiation dose of 6 Gy (XRAD 160, Precision X-Ray). The cells were incubated for a further 1 hour and were then fixed by the addition of 20 μL 3.7% formaldehyde in PBS and incubated for 20 minutes at RT. The plates were subsequently washed twice using PBS, then permeabilized with the addition of 20 μL of 0.5% Triton X-100 in PBS for 1 hour at RT. Following permeabilization, the plates were washed 2x with PBS-T and incubated with 20 μL of primary antibody (1:10,000 anti-ATM pSer1981 in PBS-T) overnight at 4°C. The next day, the plates are washed 2x with PBS-T and incubated with 20 μL of secondary antibody (1:500 AlexaFluor 488 goat anti-rabbit IgG and 1:10,000 Hoechst stock solution [10 mg/mL] in antibody buffer) for 1 hour at RT.

Following this final incubation, the plates were washed twice using PBS, sealed with black plate seals and then read on a CellInsight™ imaging platform (Thermo Scientific) with 10x objective. A two-laser setup was used to analyse nuclear staining with Hoechst (405 nm) and secondary antibody staining of pATM (488 nm).

ATR (pCHK1 Ser345)

Pharmacological activity of AZD7648 on ATR was evaluated by measuring the inhibition of 4-nitroquinoline 1-oxide (4NQO)-induced ATR phosphorylation of CHK1 (Ser345) in HT-29 cells by an imaging-based assay. HT-29 cells were dispensed into 384-well Greiner plates (#781090) to give 6000 cells/well in a total volume of 40 μL cell media and incubated overnight. Test compounds and reference controls were dosed directly into the cell plates using a Labcyte Echo 555 acoustic dispenser. The cell plates were then incubated for 1 hour at 37°C before being dosed with 3 μM of 4NQO using a Labcyte Echo 555 acoustic dispenser. The cells were incubated for a further 1 hour and were then fixed by the addition of 20 μL 3.7% formaldehyde in PBS and incubated for 20 minutes at RT. The plates were subsequently washed on a Bioteck EL406 3x using PBS-T, and then permeabilized with the addition of 20 μL of 0.1% Triton X-100 in PBS for 10 minutes at RT. Following permeabilization, the plates were washed 3x with PBST and incubated with 20 μL of primary antibody (1:250 anti-Chk1 pSer345 in PBS-T) overnight at 4°C. The next day, the plates were washed 3x with PBS and incubated with 20 μL of secondary antibody (1:500 AlexaFluor 488 goat anti-rabbit IgG and 1:5000 Hoechst stock solution [10 mg/mL] in antibody buffer) for 2 hours at RT. Following this final incubation, the plates were washed 3x with PBS, sealed with black plate seals and then read on a CellInsight imaging platform (Thermo Scientific) with 10x objective. A two-laser setup was used to analyse nuclear staining with Hoechst (405 nm) and secondary antibody staining of pChk1 (488 nm).

mTOR (pAKT Ser473)

Pharmacological activity of AZD7648 on mTOR was evaluated by measuring the inhibition of pAKT (Ser473) in MDA-MB-468 cells by an imaging-based assay. MDA-MB-468 cells were dispensed into black, 384-well Costar plates (#3712, Corning) to give 1500 cells/well in a total volume of 40 μL cell media and incubated overnight. Test compounds and reference controls were dosed directly into the cell plates using a Labcyte Echo 555 acoustic dispenser. The cell plates were then incubated for 2 hours at 37°C before being fixed by the addition of 20 μL 3.7% formaldehyde in PBS (1.2% final concentration), followed by a 30-minute RT incubation and then a 2x wash with 150 μL PBS using a

BioTek ELx406 platewasher. Cells were permeabilized and blocked with 20 μL of assay buffer (0.1% Triton X-100 in PBS + 1% BSA) for 1 hour at RT, then washed 1x with 50 μL PBS. Primary phospho-AKT (pSer473) D9E XP® rabbit monoclonal antibody (#4060, Cell Signaling Technology) was diluted 1:200 in assay buffer, 20 μL added per well, and plates were incubated at 4°C overnight. Cell plates were washed 3x with 200 μL PBS-T, then 20 μL 1:750 dilution in assay buffer of Alexa Fluor® 488 goat anti-rabbit IgG secondary antibody with a 1:5000 dilution of Hoechst was added per well. Following 1 hour of incubation at RT, plates were washed 3x with 200 μL PBS-T, and 40 μL PBS without calcium, magnesium and sodium bicarbonate (Gibco #14190-094) was added per well. Stained cell plates were covered with black seals and then read on the CellInsight imaging platform (Thermo Scientific) with a 10x objective. A two-laser setup was used to analyse nuclear staining with Hoechst (405 nm) and secondary antibody staining of pAKT (488 nm).

PI3K isoforms

PI3K selectivity of AZD7648 was evaluated in cell lines driven by different PI3K isoforms: BT474 (PI3Kα), MDA-MB-468 (PI3Kβ), RAW-264 (PI3Kγ) and JEKO-1 (PI3Kδ). Levels of phospho-AKT Thr308, a downstream target for PI3K, was used as a marker of PI3K inhibition. BT474 and MDA-MB-468 cells were seeded into 384-well plates (5600 cells/well) and incubated overnight prior to compound treatment for 2 hours. RAW-264 and JEKO-1 cells were seeded directly into 384-well plates containing compounds (1×10^5 cells/well) and incubated for 1 hour. Compounds were added to cells/plates using an Echo 555 Liquid Handler (LabCyte). BT474 and MDA-MB-468 cells were lysed in 25 μL lysis buffer (25 mM Tris-HCl, 3 mM EDTA, 3 mM EGTA, 50 mM NaF, 2 mM Na_3VO_4, 0.27 M sucrose, 10 mM β-glycerophosphate, 5 mM $Na_2P_2O_7$, 0.5% Triton X-100) for 30 minutes at RT. To stimulate AKT phosphorylation, C5a (complement 5a) anaphylatoxin trifluoroacetate salt (20 nM; Bachem) was added to RAW-264 cells for 3 minutes and anti-human IgM (120 μg/mL; Stratech) was added to JEKO-1 cells for 10 minutes prior to cell lysis. RAW-264 and JEKO-1 cells were then lysed for 30 minutes at RT. Lysates were transferred onto ELISA plates pre-coated with anti-AKT antibody (Cell Signalling #7144) that had previously been blocked with 1% BSA in PBS-T and incubated overnight at 4°C. Plates were then incubated with mouse anti-phosphoAKT Thr308 (Cell Signalling Technology #7144) for 2 hours, followed by an anti-mouse HRP-conjugated antibody (Cell Signalling Technology #7144) for 2 hours. Plates were washed between antibody incubation steps with PBS-T. QuantaBlu substrate working solution

(ThermoScientific #15169) was added to each well according to the manufacturer's instructions and stop solution was added 60 minutes later. Fluorescence was measured with an EnVision Plate Reader (Perkin Elmer) using 325 nm excitation and 420 nm emission wavelengths.

Neutral comet analysis

A548 cells that were seeded into 60 mm dishes at day 0 were DMSO or AZD7648 (1 μM) pre-treated for 30 minutes at day one. Cells were then mock radiated (no IR), radiated (2 Gy; CellRad) one ice (0h post IR) or radiated and left to recover for six hours (6h post IR) under normal growth conditions. Cells were then chilled on ice, washed once with phosphate-buffered saline (pH 7.5, lacking $CaCl_2$ and $MgCl_2$ (-/-); Gibco; PBS), scraped off and the pellets (2000 rpm, 4 °C) were resuspended in PBS (-/-) at an approximate concentration of 5 x 10^6 cells/ml. Then, 10 μL of cell suspension was mixed with 90 μL LMAgarose (37 °C; Trevigen) and 20 μL of the mixture was spotted onto GelBond Film (Lonza), covered by a 12 mm round cover glass (VWR) and incubated at 4 °C for 5 minutes. Upon cover glass removal, the samples were incubated in pre-chilled lysis solution (Trevigen) for one hour at 4 °C. Lysed samples were incubated in chilled (4 °C) neutral electroforesis buffer (50 mM Tris Base, 150 mM Sodium acetate, pH 9.0)) for 30 minutes and then exposed to a 21 V current for 45 minutes in chilled neutral electroforesis buffer at room temperature (RT). Samples were fixed in 70% (v/v) ethanol for 10 minutes, dried over night and stained with SYBER green nucleic acid staining solution (Invitrogen) in Tris-EDTA (TE; pH 7.5) buffer for 10 minutes at RT. Samples were washed in ultra pure water, images were automatically aquired with a Leice inverted microscope and neutral comets were analysed with Comet4 imaging software. Per condition, tail lenghts of 50 individual cells were quantified and plotted.

High-content immunofluorescence imaging and analysis

A549 or OAW42 cells were plated 1000 cells/well into Cell Carrier Ultra 384-well plates (Perkin Elmer) in 40 μL and incubated overnight. AZD7648 and reference controls were dosed by acoustic dispensing using the Echo 555 Liquid Handler (LabCyte). Cell plates were then incubated for 1 hour at 37°C before receiving 2 Gy irradiation (XRAD 160, Precision X-Ray) for A549 cells or doxorubicin (Sigma D1515) treatment for OAW42 cells. Cells were then incubated for the indicated time points and fixed with 4% paraformaldehyde for 30 minutes at RT, followed by a wash with PBS using a Biotek EL406 system. Fixed cells were permeabilized and blocked for 1 hour at RT with 1% BSA in PBS + 0.1% Triton X-100. For staining, blocking solution was then removed and 20 μL/well of anti-γH2AX, anti-53BP1 and anti-ATM pSer1981 antibodies at 1:10,000

dilution in PBS + 0.1% Triton X-100 was added and incubated overnight at 4°C. Plates were then washed 2x with PBS, incubated with 20 μL of antibody solution containing 1:1000 Hoechst and 1:500 dilution of each of the Alexa Fluor® secondary antibodies, and incubated at RT for 2 hours. Plates were washed 3x with PBS before imaging on the CV7000 high-content imaging platform (Yokogawa). Following image acquisition, images were exported to the Columbus™ image data storage and analysis system (Perkin Elmer). Optimized image algorithms were used to evaluate phenotypic responses using nuclear staining with Hoechst (cell cycle, micronuclei, nuclear fragmentation), as well as to quantify the response of DNA damage response markers (foci numbers and total signal of ATM pSer1981, γH2AX and 53BP1 staining).

SDS-PAGE and Western blotting of samples

Protein lysates were generated by scraping cells in radioimmunoprecipitation assay (RIPA) lysis buffer (ThermoFisher) supplemented with 1% sodium dodecyl sulfate (SDS) and protease and phosphatase inhibitor tablets (Roche) following compound treatments at indicated time points. Protein concentration was determined using the BCA protein assay kit (ThermoFisher). Proteins were separated by SDS-PAGE on NuPAGE® 4–12% Bis-Tris 3–8% Tris-acetate gels (ThermoFisher) and transferred onto nitrocellulose membranes using the iBlot® dry blotting system (ThermoFisher). Membranes were blocked with 5% milk or BSA in Tris-buffered saline (TBS) with 0.05% Tween-20 (TBS-T) and incubated with primary antibody overnight at 4°C. Membranes were then washed with TBS-T and incubated with HRP-conjugated anti-rabbit or anti-mouse antibodies for 1 hour at RT. Proteins were detected by incubating membranes with SuperSignal™ Dura extended-duration substrate (ThermoFisher) and visualized using the G:BOX ChemiGenius imaging system (Syngene). Band intensities were quantified using Syngene Genetools software. For analysis of *in vivo* samples, a two-sided t-test was performed on logged data assuming unequal variance. A list of all antibodies used in this study can be found in Supplementary Table 5.

Colony formation assay

Cells were plated at low density into six-well plates in 3 mL medium (500 cells for A549, NCI-H1299 and FaDu WT, 4000 cells for FaDu ATM WT) and incubated overnight. Cell plating numbers were adjusted for higher IR doses to compensate for cell kill. Cells were treated with three radiation doses (1–4 Gy) using a Faxitron CellRad irradiator (130 kV, 5 mA, 0.5 mm Al) ± 1 hour pre-treatment with AZD7648 at indicated concentrations for

8–14 days or with increasing concentrations of olaparib. Cell colonies were washed with PBS and stained with Brilliant Blue solution (Sigma) containing 50% methanol and 7% acetic acid for >10 minutes. Excess stain was removed by washing with water. Surviving colonies (>50 cells) were counted using the GelCount™ colony counter (Oxford Optronix). Mean surviving colonies were calculated from three technical replicates. Surviving fraction was calculated relative to the vehicle (DMSO) or AZD7648-alone-treated controls.

For the IR combination treatment experiments, the relative number of surviving colonies for each treatment condition was normalized for plating efficiency at each radiation dose before survival fraction was calculated. The radiation dose–response curve was fitted to the linear quadratic model ($y=\exp[-\alpha \times x-\beta \times x^2]$)[1]. The dose enhancement factor (DEF_{37}) was calculated by dividing the radiation dose required in the presence of the compound by the radiation dose required in the absence of compound to achieve 37% surviving fraction. For the olaparib treatment experiments, the concentration–response curve was fitted to a four-parameter non-linear regression model using GraphPad Prism software. The IC_{50} is the concentration required to give a 50% reduction in the relative surviving fraction.

Live/Dead assay

Cells were plated into 384-well plates in 70 μL medium at appropriate cell densities for a 5- to 7-day treatment period, allowing at least three doublings to occur during this time. Next day, cells were treated with a 5x5 compound concentration matrix of AZD7648 (0.1 – 10 μM) and doxorucibin (0.003 – 0.32 μM) or olaparib (0.1 – 10 μM) at semi-log dilutions using the Echo 555 Liquid Handler (LabCyte) for 5–7 days. Dead cells were identified by staining cells with SYTOX® Green nucleic acid stain solution (1:2500 in 5 mM EDTA in TBS; ThermoFisher) for 1.5 hours at RT in the dark and quantified using the Acumen® Cellista (TTP Labtech). Next, the total number of cells was determined by staining cells with Saponin solution (0.25% in 5 mM EDTA in TBS; Sigma) for 16 hours at RT in the dark and quantified using the Acumen® Cellista (TTP Labtech). Dead and total cell numbers were also determined on the day of treatment (day 0). These data were used for combination synergy score analysis. For OAW42 cells, the doxorubicin response curve ± AZD7648 was fitted to a four-parameter non-linear regression model using GraphPad Prism software.

CellTiter-Glo assay

HCC70 cellular response to compound treatment was measured by CellTiter-Glo luminescent viability assay (Promega) rather than Live/Dead assay as their cell morphology made them challenging to accurately determine cell numbers by Live/Dead assay. HCC70 cells were treated with the same method as described for the Live/Dead assay. Cells were then incubated with 35 μL of CellTiter-Glo reagent (Promega) and incubated at RT for 12 minutes. Luminescence was measured on a SpectraMax i3x microplate reader (Molecular Devices). Luminescence measurements were also taken on the day of treatment (day 0). These data were used for combination synergy score analysis.

Combination synergy score analysis

A synergy score was determined for the effect of compound combination treatments using data generated from the Live/Dead and CellTiter-Glo assays with Genedata Screener® software. For the Live/Dead assay, live cell number was calculated by subtracting the number of dead cells from the total number of cells. Percentage growth inhibitory activity was determined by calculating the live cell number after compound treatment relative to the live cell number at day 0. For HCC70 cells, percentage growth inhibitory activity was determined by calculating the luminescence measured after compound treatment relative to the luminescence measured at day 0. An excess score was determined, reflecting the extent to which the observed effect was greater than the predicted additive effect of combining the two compounds based on the Loewe additivity model[2]. The synergy score was derived from the excess score taking into account the compound concentration dependency. A score of >0 is indicative of an additive combination effect and ≥5 is indicative of synergistic combination activity.

Cell confluency assay

Cells were plated into 96-well plates in 100 μL medium (1000 cells for FaDu WT, 1500 cells for FaDu ATM KO, 250 cells for A549 WT and A549 ATM KO) and incubated overnight. Cells were treated with AZD7648 and/or olaparib at indicated concentrations using an HP D300 digital dispenser (Tecan). Cell confluency was determined from four fields of view per well every 12 hours over a period of 10 days using an IncuCyte® ZOOM live cell analysis imaging system (Essen Bioscience). Data represent mean cell confluency of duplicate wells.

Cell cycle analysis by flow cytometry

FaDu ATM WT or ATM KO cells were plated into six-well plates (1 x 10^5 cells/well) and incubated overnight. Cells were treated with AZD7648 or olaparib at indicated concentrations for 48 hours. The CHK1 inhibitor LY2606368 (70 nM) was added 2 hours before cells were collected. Medium was collected and cells were washed with PBS. PBS was collected and cells were detached using TrypLE™ Express Enzyme (Gibco). The collected medium, PBS and cells were centrifuged (1200 rpm, 5 minutes) and the cell pellet was re-suspended in 50 μL PBS. One millilitre ice-cold 70% ethanol was added dropwise to the cell suspension over a vortex at medium speed and stored at –20°C overnight. Fixed cells were washed in 5 mL antibody-blocking buffer (PBS containing 1% BSA) and centrifuged (1200 rpm, 5 minutes) twice. Cells were stained with DAPI (1 μg/mL in antibody-blocking buffer) and DNA content was measured on a FACS Aria II flow cytometer (BD Biosciences). Single-cell populations were gated and a total of 10,000 cell events were acquired. Percentage cell cycle distribution was determined using FlowJo software. Representative gating strategy used can be seen in Supplementary Figure 13. Significant differences were calculated by Student t-test.

Metaphase spread analysis

FaDu ATM WT or ATM KO cells were seeded into 6 cm dishes (7 x 10^5 cells/well in 4 mL) in normal growth medium. Cells were immediately vehicle (DMSO) or compound treated for 48 hours. Forty-three hours post-treatment, cells were incubated with 30 ng/mL Colcemid (KaryoMAX® Colcemid™ solution in PBS; ThermoFisher) for 5 hours. Medium was collected and cells were washed once with PBS (Gibco). PBS was collected and cells were detached from the plate using TrypLE™ Express reagent (Gibco). Upon detachment, cells were re-suspended, collected and centrifuged at 300g for 5 minutes at RT. Supernatant was removed and the tubes were flicked to loosen the cell pellet. Cells were then re-suspended in 10 mL pre-warmed (37°C) hypotonic solution (0.075 M KCl; Gibco) and incubated at 37°C for 12 minutes. One millilitre ice-cold fixative (methanol [VWR] and acetic acid [Sigma]; 3:1 ratio) was added and mixed into the cell suspension to avoid clumping of the cells. The tubes were then centrifuged at RT for 8 minutes (400g), supernatants were removed, tubes were flicked, and to every tube 5 mL ice-cold fixative was added dropwise while carefully swirling the cells. The tubes were centrifuged at RT for 8 minutes (400g), supernatants were removed, tubes flicked and 5 mL fixative/tube was added along the tube wall. The metaphase preparations were then incubated overnight at –20°C and the pellets were washed a further 3x as described above.

Depending on the number of cells, up to 90% of the fixative was removed to ensure correct cell density prior to dropping. Using a plastic Pasteur's pipette, two to three droplets of cell suspension were dropped onto a microscope slide (Superfrost™ Plus and ColorFrost™ Plus microscope slides; VWR). The slides were then left to dry and stained with 8% Giesma (Sigma; in buffered water [pH 6.8]). The stained slides were washed carefully but thoroughly with water and left to dry overnight. Cover slips were then mounted onto the microscope slides using DPX mountant (Sigma #44581) and left to dry overnight at RT. Using the Metafer 4 instrument (Metasystems), up to 1000 metaphase spreads were located on the slide, chromosomal aberrations described as chromatid or chromosome breaks, and chromosome fusions were scored by eye for 50 metaphase spreads per sample.

Caspase activity assay

FaDu WT cells were plated at 1000 cells/well and FaDu ATM KO at 1500 cells/well in 96-well plates. Medium was replaced next day with 100 μL medium containing IncuCyte® Caspase 3/7 Green apoptosis reagent (5 μM final concentration; Essen Bioscience). Cells were treated with AZD7648 and/or olaparib at indicated concentrations using an HP D300 digital dispenser (Tecan). Cells were imaged using an IncuCyte® live cell analysis system (Essen Bioscience) for real-time apoptosis detection. Four images per well were taken every 3 hours, using a 10x objective with phase contrast and green fluorescence channels. Data analysis was performed using the IncuCyte® ZOOM integrated software. Mean fluorescence levels were normalized to the total cell confluency and then calculated relative to DMSO vehicle-treated control. Significant differences were calculated by Student t-test.

***In vivo* studies**

Xenografts of A549, H1299, BT474, FaDu ATM KO and FaDu WT human cells (CDX) were established by implantation of 100 μL of a cell suspension subcutaneously into the dorsal left flank of female nude or severe combined immunodeficient (SCID) mice of at least 6 weeks of age. The day prior to the BT474 inoculation, the mice were implanted subcutaneously into the right dorsal flank with a slow-release oestrogen pellet. For HBCx-17[3], OV2022, CTG-703, CTG-0828 and CTG-0149 PDX studies, female athymic nude or SCID mice were surgically implanted subcutaneously with tumour fragments from donor mice. Tumours were measured (length x width) by bilateral Vernier calliper measurements and tumour volume calculated using Mousetrap software. Mice were randomized into

treatment groups when mean tumour volume reached ~0.2 cm^3. AZD7648 was formulated in 0.5% hydroxypropyl methylcellulose (HPMC)/0.1% Tween80 (HPMC/T) and orally dosed (4–100 mg/kg). When dosed twice daily (bid), the time between the morning and evening doses was 8 hours. Targeted irradiation of 2 Gy was delivered over 2 minutes daily over the first 5 days of treatment. Liposomal doxorubicin (Doxoves) was diluted in physiological saline and intravenously dosed at 2.5 or 5 mg/kg once per week. Olaparib was formulated in 10% DMSO/30% Kleptose and orally dosed at 100 mg/kg once daily. All three combinations were dosed 1 hour after the morning dose of AZD7648 or its vehicle HPMC/T. Tumour growth inhibition from start of treatment was assessed by comparison of the mean change in tumour volume for the control and treated groups, using the Mousetrap application and represented as tumour growth inhibition (TGI). Statistical significance was evaluated using a one-tailed t-test. HBCx-17 PDX study was carried out at XenTech, France in accordance with French regulatory legislation concerning the protection of laboratory animals. CTG-703, CTG-0828 and CTG-0149 PDX studies were carried out at Champions Oncology, Inc., USA in accordance to the guidelines of the Institutional Animal Care and Use Committee (IACUC) of Champions Oncology and the USA regulatory legislation. CDX and OV2022 PDX studies were conducted in the UK in accordance with UK Home Office legislation, the Animal Scientific Procedures Act 1986, and AstraZeneca's global bioethics policy. Experimental details are outlined in Home Office project licences 70/8839, 70/8894 and P0EC1FFDF.

Pharmacokinetic analysis

Blood samples were taken via venipuncture of the tail vein. Whole blood was mixed 1:5 with PBS and centrifuged at 1500g for 3 minutes at 4°C, and the plasma was extracted and frozen at –80°C. To assess plasma levels of AZD7648, each plasma sample (25 μL) was prepared using an appropriate dilution factor and compared against an 11-point standard calibration curve (1–10,000 nM) prepared in DMSO and spiked into blank plasma. Acetonitrile (100 μL) was added with the internal standard, followed by centrifugation at 3000 rpm for 10 minutes. Supernatant (50 μL) was then diluted in 300 μL water and analysed with ultra-performance liquid chromatography–tandem mass spectrometry.

Xenograft sample preparation

Animals were euthanized and tumour samples snap frozen in liquid nitrogen or fixed in formalin and paraffin embedded for subsequent analysis. Tumour pieces were lysed in

600 μL of ice-cold buffer containing Tris–NaCl pH 7.5 20 mmol/L, NaCl 137 mmol/L, glycerol 10%, supplemented with NaF 50 mmol/L, Na_3VO_4 1 mmol/L, protease complete inhibitor tablet (Roche 1836145), Benzonase nuclease 1 μL per 5 mL (Sigma-Aldrich E1014-5KU), phosphatase inhibitor cocktails 2 and 3 (Sigma-Aldrich, P0044 and P5726), and protease inhibitor cocktail (Sigma P8340). Homogenization was performed 2x using Fastprep tubes (MP Biomedicals #6910-500) and an MP Biomedicals Fast Prep-24 machine. All samples were sonicated for 30 seconds at high amplitude (Diagenode) and diluted further with 400 μL of lysis buffer containing 2.5% SDS and 2.5% NP40 (Roche), so that final concentration of each detergent was 1%, to allow protein to be released from DNA (this was not added before sonication to avoid foaming). Samples were then centrifuged at 13,000 rpm and 4ºC for 10 minutes, and the supernatants were collected.

Immunohistochemistry

For IHC staining, tissues were dewaxed in xylene, rehydrated in graded alcohol and water, and antigen retrieved at 110°C with Dako retrieval buffer (pDNA-PKcs: pH 6, 5 minutes; γH2AX: pH 9, 2 minutes). The LabVision autostainer (Thermo Scientific) was used for staining; slides were incubated in 3% hydrogen peroxide for 10 minutes, serum-free protein block (Dako) for 20 minutes, primary antibody in TBS-T for 60 minutes, EnVision™ + System-HRP labelled polymer (rabbit; Dako) for 30 minutes, and diaminobenzidine for 10 minutes (Dako). Washes were done with TBS-T. Carazzi's haematoxylin was used to counterstain the nuclei.

Supplementary References

1. Brenner DJ. The linear-quadratic model is an appropriate methodology for determining isoeffective doses at large doses per fraction. *Semin Radiat Oncol* **18**, 234-239 (2008).
2. Foucquier J, Guedj M. Analysis of drug combinations: current methodological landscape. *Pharmacol Res Perspect* **3**, e00149 (2015).
3. de Plater L*, et al.* Establishment and characterisation of a new breast cancer xenograft obtained from a woman carrying a germline BRCA2 mutation. *Br J Cancer* **103**, 1192-1200 (2010).
