# Supplementary material for: AZD7648 is a potent and selective DNA-PK inhibitor that enhances radiation, chemotherapy and olaparib activity
Source: Nat Commun. 2019 Nov 7;10:5065. doi: 10.1038/s41467-019-12836-9 (PMC6838110; doi:10.1038/s41467-019-12836-9)
Supplement: Supplementary file 2 — Reporting Summary [file 41467_2019_12836_MOESM2_ESM.pdf]

nature research

Corresponding author(s): Elaine Cadogan

Last updated by author(s): Sep 11, 2019

Reporting Summary

Nature Research wishes to improve the reproducibility of the work that we publish. This form provides structure for consistency and transparency in reporting. For further information on Nature Research policies, see Authors & Referees and the Editorial Policy Checklist.

Statistics

For all statistical analyses, confirm that the following items are present in the figure legend, table legend, main text, or Methods section.

n/a	Confirmed	
☐	☒	The exact sample size (n) for each experimental group/condition, given as a discrete number and unit of measurement
☐	☒	A statement on whether measurements were taken from distinct samples or whether the same sample was measured repeatedly
☐	☒	The statistical test(s) used AND whether they are one- or two-sided<br>*Only common tests should be described solely by name; describe more complex techniques in the Methods section.*
☐	☒	A description of all covariates tested
☐	☒	A description of any assumptions or corrections, such as tests of normality and adjustment for multiple comparisons
☐	☒	A full description of the statistical parameters including central tendency (e.g. means) or other basic estimates (e.g. regression coefficient) AND variation (e.g. standard deviation) or associated estimates of uncertainty (e.g. confidence intervals)
☐	☒	For null hypothesis testing, the test statistic (e.g. F, t, r) with confidence intervals, effect sizes, degrees of freedom and P value noted<br>*Give P values as exact values whenever suitable.*
☒	☐	For Bayesian analysis, information on the choice of priors and Markov chain Monte Carlo settings
☒	☐	For hierarchical and complex designs, identification of the appropriate level for tests and full reporting of outcomes
☒	☐	Estimates of effect sizes (e.g. Cohen's d, Pearson's r), indicating how they were calculated

Our web collection on statistics for biologists contains articles on many of the points above.

Software and code

Policy information about availability of computer code

Data collection	*Provide a description of all commercial, open source and custom code used to collect the data in this study, specifying the version used OR state that no software was used.*
Data analysis	For the analysis of the immunofluorescence images dataset, we used the commercially available Columbus image data storage and analysis system (Perkin Elmer).

For manuscripts utilizing custom algorithms or software that are central to the research but not yet described in published literature, software must be made available to editors/reviewers. We strongly encourage code deposition in a community repository (e.g. GitHub). See the Nature Research guidelines for submitting code & software for further information.

Data

Policy information about availability of data

All manuscripts must include a data availability statement. This statement should provide the following information, where applicable:

- Accession codes, unique identifiers, or web links for publicly available datasets
- A list of figures that have associated raw data
- A description of any restrictions on data availability

All the associated raw data for our Figures can be available upon request. We have included a data availability statement in the main manuscript.

Field-specific reporting

Please select the one below that is the best fit for your research. If you are not sure, read the appropriate sections before making your selection.

☒ Life sciences ☐ Behavioural & social sciences ☐ Ecological, evolutionary & environmental sciences

Life sciences study design

All studies must disclose on these points even when the disclosure is negative.

Sample size	For in vivo studies, group sizes were determined using power calculation based on historical growth data in the group, for the models. For the in vitro studies, we chose sample sizes based on what is generally accepted and published on those particular types of assays.
Data exclusions	No data were excluded.
Replication	Most of the data presented was repeated more than twice. All attempts on replication show reproducibility of the data presented.
Randomization	For in vivo experiments, animals were randomised at selection for the live phase. For the PD analysis, samples were randomised prior to Western blotting. In vitro studies were not randomised, but appropriate controls were run.
Blinding	N/A

Reporting for specific materials, systems and methods

We require information from authors about some types of materials, experimental systems and methods used in many studies. Here, indicate whether each material, system or method listed is relevant to your study. If you are not sure if a list item applies to your research, read the appropriate section before selecting a response.

Materials & experimental systems

n/a	Involved in the study	
☐	☒	Antibodies
☐	☒	Eukaryotic cell lines
☒	☐	Palaeontology
☐	☒	Animals and other organisms
☒	☐	Human research participants
☒	☐	Clinical data

Methods

n/a	Involved in the study	
☒	☐	ChIP-seq
☐	☒	Flow cytometry
☒	☐	MRI-based neuroimaging

Antibodies

Antibodies used	A full list of antibodies and their details can be found in Supplementary Table 5.
Validation	All antibodies used this study have been validated by the manufacturer and/or been validated in publications.

Eukaryotic cell lines

Policy information about cell lines

Cell line source(s)	A full list of cell lines and their source are listed in Supplementary Table 4.
Authentication	All cell lines were authenticated by STR testing by AstraZeneca.
Mycoplasma contamination	All cell lines were tested negative for mycoplasma contamination by AstraZeneca.
Commonly misidentified lines (See ICLAC register)	N/A

Animals and other organisms

Policy information about studies involving animals; ARRIVE guidelines recommended for reporting animal research

Laboratory animals	All the details on mouse models are described in the manuscript.
Wild animals	N/A
Field-collected samples	N/A
Ethics oversight	All work was done under project license 70/8839, 70/8894 and P0EC1FFDF granted by the Home Office of the United Kingdom.

Note that full information on the approval of the study protocol must also be provided in the manuscript.

Flow Cytometry

Plots

Confirm that:

☒ The axis labels state the marker and fluorochrome used (e.g. CD4-FITC).

☒ The axis scales are clearly visible. Include numbers along axes only for bottom left plot of group (a 'group' is an analysis of identical markers).

☒ All plots are contour plots with outliers or pseudocolor plots.

☒ A numerical value for number of cells or percentage (with statistics) is provided.

Methodology

Sample preparation	See section "Cell cycle analysis by flow cytometry" in Material and Methods Section in the Supplementary Information
Instrument	See section "Cell cycle analysis by flow cytometry" in Material and Methods Section in the Supplementary Information
Software	See section "Cell cycle analysis by flow cytometry" in Material and Methods Section in the Supplementary Information
Cell population abundance	Section "Cell cycle analysis by flow cytometry" in Material and Methods Section in the Supplementary Information
Gating strategy	Total cell population was gated using FSC-H (x axis) and SSC-H (y axis). Single cell populations were gated using DAPI area (x axis) and width (y axis). Doublets (where area and width are disproportional to each other) were excluded, but >4N cells were included. Cell cycle distribution was observed by plotting DAPI-A against cell number. Cell cycle gating was carried out based on the width of the peaks of the cell cycle profile.

☒ Tick this box to confirm that a figure exemplifying the gating strategy is provided in the Supplementary Information.
